# The Burden and Risk Factors of Gastric Cancer in Eastern Asia From 1990 to 2021: Longitudinal Observational Study of the Global Burden of Disease Study 2021

**DOI:** 10.2196/75728

**Published:** 2025-08-08

**Authors:** Weijia Kong, Yuting Sun, Xiaoyan Qin, Guanghui Zhu, Xiaoyu Zhu, Ziyu Kuang, Zhigang Xiao, Jie Li

**Affiliations:** 1 Guang’anmen Hospital, China Academy of Chinese Medical Sciences, 5 Beixiange Street, Xicheng District, Beijing, 10053, China, +86-10-83123311; 2 Graduate School, Beijing University of Chinese Medicine, Beijing, China

**Keywords:** gastric cancer, risk factors, mortality, age-period-cohort analysis, joinpoint analysis

## Abstract

**Background:**

Eastern Asia has historically had the highest global incidence and mortality rates of gastric cancer (GC) while substantial disparities exist between countries. The overall burden of GC remains insufficiently explored.

**Objective:**

Using the Global Burden of Disease Study 2021, this research aims to estimate the burden and risk factors of GC in Eastern Asia from 1990 to 2021.

**Methods:**

Incidence, age-standardized incidence rate (ASIR), deaths, age-standardized mortality rate (ASMR), disability-adjusted life years, age-standardized disability-adjusted life year rate (ASDR), and risk factor burdens for GC were analyzed in Eastern Asia from 1990 to 2021. Joinpoint analysis determined average annual percent change (AAPC) and annual percent change, while age-period-cohort analysis assessed temporal trends. The Bayesian age-period-cohort model projected GC burden from 2021 to 2035. All analyses used R software (version 4.4.1; R Foundation for Statistical Computing).

**Results:**

In 2021, Eastern Asia reported 748,235 new GC cases and 527,054 deaths, accounting for 60.8% (748,235/1,230,232) of new cases and 55.2% (527,054/954,373) of deaths reported globally. From 1990 to 2021, South Korea showed the largest declines in ASIR, ASMR, and ASDR, with ASMR decreasing from 55.4 per 100,000 to 13.3 per 100,000 (AAPC −4.5, 95% CI −4.8 to −4.3). ASIR, ASMR, and ASDR also showed a downward trend in Japan and China, with an AAPC of −3.0 (95% CI −3.2 to −2.8) for ASMR in Japan and −2.4 (95% CI −2.6 to −2.3) in China. The GC burden of North Korea was basically stable, with an AAPC of ASMR of −0.8 (95% CI −0.8 to −0.8). Mongolia showed a slight decline, with an AAPC of ASMR of −1.4 (95% CI −1.7 to −1.0), and the burden of GC was the highest. High-sodium diets and smoking were the main risk factors for disability-adjusted life years of GC in 2021. Smoking contributed to a decline in ASDR as the sociodemographic index increased. Projections suggest continued ASDR reductions across Eastern Asia from 2022 to 2035, though Mongolia will maintain the highest burden.

**Conclusions:**

Despite a decrease from 1990 to 2021, GC remains a significant public health issue in Eastern Asia. Addressing it necessitates prioritizing primary and secondary prevention, including reducing risk factors and enhancing early screening.

## Introduction

According to the latest global cancer burden figures for 2022 released by the World Health Organization’s (WHO) International Agency for Research on Cancer (IARC), gastric cancer (GC) is the fifth leading cause of death with the fifth most common cancer globally [1]. There were over 968,000 new cases of GC in 2022 and close to 660,000 deaths. Although the incidence and mortality of GC present a downward trend worldwide [2], incidence and mortality rates still are highest in Eastern Asia [1]. However, there is a large variability across Eastern Asia countries in both incidence and mortality, which might be associated with socioeconomic disparities, early GC screening, etc.

The risk of infection with *Helicobacter pylori*, a major confirmed cause of noncardia GC, is enhanced by poor hygiene; thus, the incidence of GC is closely related to socioeconomic differences [3]. A recently published cluster randomized controlled trial with 11.8 years of follow-up from China showed a reduction in the incidence of GC among patients treated with anti*–H. pylori* [4]. In addition, smoking, heavy alcohol consumption, high-sodium diet, and insufficient intake of fresh vegetables and fruits may also increase the risk of GC [5]. In addition, because of the insidious symptoms in early GC, a high proportion of patients are already progressed into middle or late stage by the time it is detected, and the prognosis is poor with a 5-year survival rate of <25% [6]. However, due to the widespread implementation of national screening programs, some high-incidence Eastern Asia countries have shown relatively high 5-year survival rates for GC through early cancer detection (such as 64.6% in Japan and 71.5% in South Korea) [7]. Therefore, the comparison of the trend features of GC in Eastern Asia countries with a heavy burden of GC is helpful to reveal the epidemiological characteristics and risk factors of GC and provide information support for the formulation of public health policies.

In this study, we used Eastern Asia data from the Global Burden of Disease Study (GBD) 2021 to explore the changes in GC burden and its risk factors by sex and nation from 1990 to 2021. The findings aim to offer scientific evidence for the accurate screening and prevention of GC, ultimately reducing its disease burden.

## Methods

### Ethical Considerations

This study uses publicly available data from the Global Burden of Disease Study (GBD) database. The GBD data are anonymized and aggregated at the population level, and no individual-level information is included. Ethics approval and informed consent were not required for the use of these data. Access to the GBD database complies with all applicable data use agreements and guidelines provided by the Institute for Health Metrics and Evaluation [8].

### Overview and Definition

GBD 2021 assesses health risks associated with 371 diseases, injuries, and 88 risk factors, and we conducted a secondary analysis of data from this collaborative endeavor. This study complies with the GATHER (Guidelines for Accurate and Transparent Health Estimates Reporting) checklist and has been registered in the GBD 2021 Paper Proposal Form. This analysis included patients with GC stratified by sex and age in Eastern Asia (containing 5 countries) from 1990 to 2021. Since GBD 2021 excludes data on the GC burden for individuals younger than 15 years of age, our main focus was on those aged 15 years and older. GC was classified according to the *International Classification of Diseases* (*ICD*), including *ICD-10* codes C16-C16.9 and *ICD-9* codes 151‐151.9, 209.23, version 10.04 [9].

Data on the incidence, mortality, and disability-adjusted life years (DALYs) for GC in Eastern Asia, including their 95% uncertainty intervals (UIs), were obtained from GBD 2021. Eastern Asia population projections from 2022 to 2035 were also derived from the 2021 Global Health Data Exchange source tool. Sociodemographic index (SDI) is a comprehensive index to measure the socioeconomic development of a region. In this study, Eastern Asia was classified according to the quintile of SDI. The Institute for Health Metrics and Evaluation offers the regional SDI division [10]. The GBD 2021 companion paper explains the data input, processing, synthesis, and the final model used to estimate disease burden [11]. The estimates referenced in this study are publicly accessible through the GBD results tool [12].

### Attributable Risk Factors

For the risk factors of GC in GBD 2021, exposure data were modeled using a Bayesian statistical model: either spatiotemporal Gaussian process regression or DisMod-MR 2.1.9 to estimate the exposure distribution of risk factors. Next, the general formula for continuous risk was used to compute the Population Attributable Fraction (PAF) between risk-outcome pairs by age-sex-location-year. To estimate attributable DALYs, the total DALY was multiplied by the PAF. Previous studies detail the exact calculation process [9]. Risk factors associated with GC in GBD 2021 include smoking and high-sodium diet. We showed shifts in the PAF for these risk factors between 1990 and 2021 and used locally weighted regression to explore the relationship between risk factors and SDI.

### Statistics

#### Age-Standardized Incidence, Mortality, and DALY Estimates

The age-standardized incidence rate (ASIR), age-standardized death rate (ASMR), and age-standardized disability-adjusted life year rate (ASDR) were adopted to quantify the trends associated with morbidity, mortality, and DALYs for the all-age population in Eastern Asia. In this study, the direct methods were used to calculate age-standardized rates (per 100,000 population) [13], in which the actual rates of all groups of comparative data are known, and the rates are standardized by standard population or standard population composition. Standardization removes the effect of internal differences (such as age) and subsequently allows analysis of any substantial differences. Due to GBD 2021 only offering crude rates for specific age groups (eg, 15‐19, 20‐24, 25‐29, and up to 95+ years), we used crude rates for such groups. All data are accessible on the web via the GBD results tool [12].

#### Joinpoint Analysis

In this study, we used the Joinpoint software (version 4.9.1.0) developed by the National Cancer Institute Division of Cancer Control & Population Sciences to calculate annual percent change (APC), average annual percent change (AAPC), and its 95% CI of the ASIR, ASMR, and ASDR of GC in Eastern Asia. The Joinpoint software used the Monte Carlo permutation test model optimization method to select the optimal number of joinpoints. Our data used the optimal number of joinpoints recommended by the software for analysis.

Joinpoint regression (selecting the log-linear model: lny=xb), also known as segmented line regression, is a statistical method to analyze trend changes in time series data. This method calculates the APC and its 95% CI for each segment by dividing the long-term trend line into a number of segments to describe the time series trend by sex in Eastern Asia countries. Based on the trend of the APC, the AAPC was then computed. An upward trend is indicated when both the APC or AAPC estimate and its 95% CI upper and lower bounds are greater than 0. Alternatively, a downward trend occurs if both the APC or AAPC estimate and its 95% CI upper and lower bounds are less than 0.

#### Age-Period-Cohort Analysis

We used age-period-cohort modeling based on the Poisson distribution to assess the connections between age, period, and birth cohort with GC mortality [14], as an individual’s birth cohort is evaluated using the time period of death and the individual’s death age (birth cohort=period-age).

However, the age-period-cohort analysis experiences unidentifiable issues, so we used the intrinsic estimation method to estimate the age, period, and cohort effects. These estimates were validated with the use of the estimation function of the age-period-cohort network analysis tool developed by the National Cancer Institute.

The estimable functions contained the longitudinal age curve, period or cohort relative risk (RR), and net or local drift. The longitudinal age curve shows the expected age-specific rate in a reference cohort adjusted for period effects. The period or cohort RR would be the relative death risk after being adjusted for age and nonlinear effects in a period or cohort compared with the reference period or cohort. Net and local drift indicate APC in all age groups and each age group, respectively.

#### Bayesian Age-Period-Cohort Analysis

For projecting future disease burden, the Bayesian age-period-cohort (BAPC) model provides the highest coverage and greater accuracy compared to other models [15,16]. The age-period-cohort model was used to assess the age, period, and cohort effects on GC mortality in Eastern Asia, and the BAPC model was used to estimate GC mortality from 2021 to 2035. The forecast was conducted using the BAPC model within the integrated nested Laplacian approximation (R packages BAPC and INLA) to adjust for overdispersion by assuming an inverse γ prior distribution for age, period, and cohort effects (modeled by a second-order random walk [RW2]) [17] .

The R software package (version 4.4.1; R Foundation for Statistical Computing) and JD_GBDR (version 2.22; Jingding Medical Technology Co, Ltd) were used for graphical drawing in this study. A 2-sided *P* value of .05 or less was used to define statistical significance.

## Results

### Burden of GC in 2021

**Figure 1.**
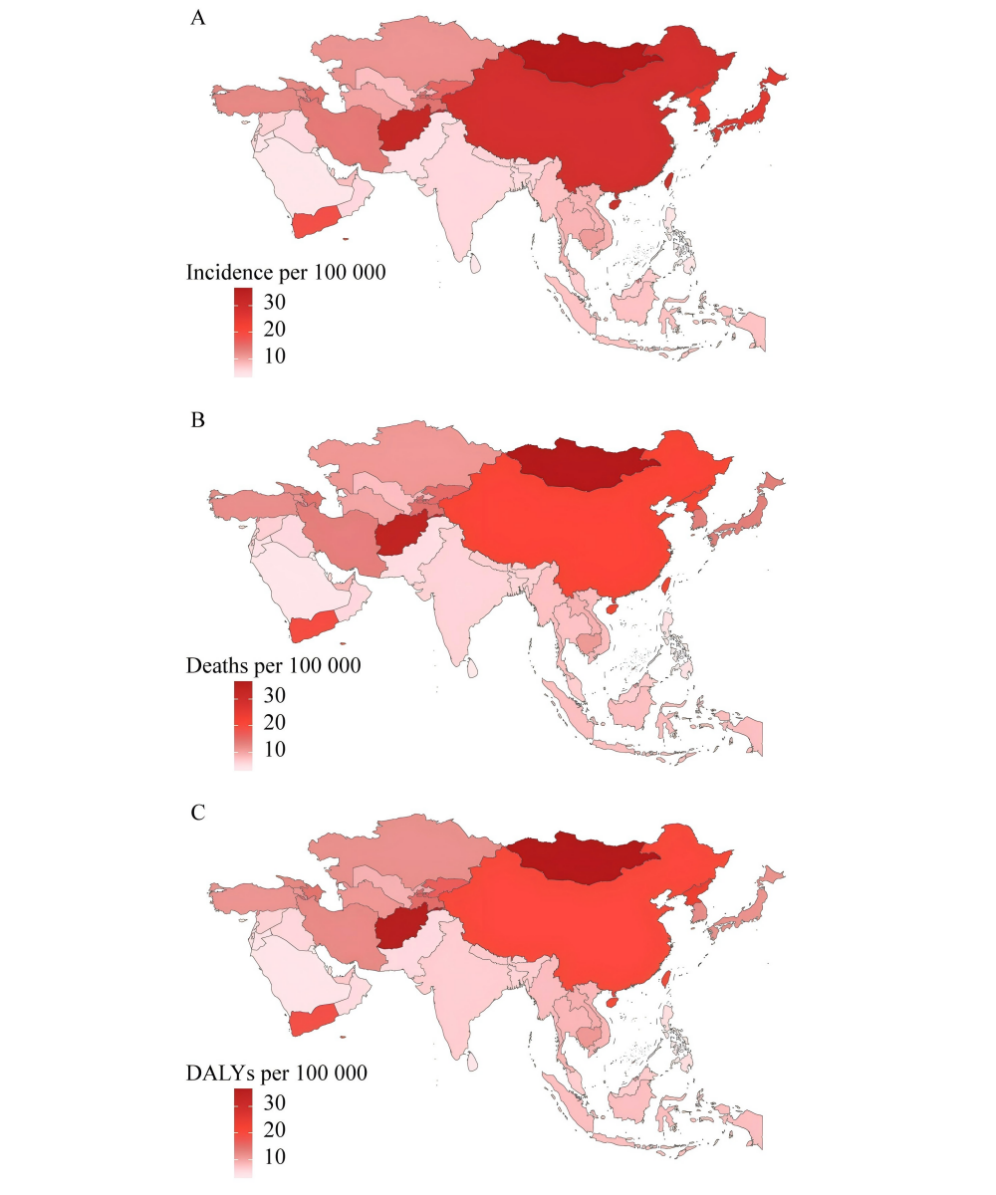
Age-standardized (A) incidence, (B) deaths, and (C) DALY rate of gastric cancer in Asia in 2021. DALY: disability-adjusted life year.

In 2021, there were 748,235 new cases of GC and 527,054 deaths in Eastern Asia, with DALYs of 12.1 million, accounting for 60.8% (748,235/1230,232) new cases and 55.2% (527,054/954,373) deaths reported globally. Among the countries in Eastern Asia, the ASIR for GC was highest in Mongolia (36.8, 95% UI 29.4-45.3 per 100,000), the second was China (29.1, 95% UI 22.4-36.2 per 100,000), followed by South Korea, Japan, and North Korea (Figure 1A and Multimedia Appendix 1). The ASMR for GC was highest in Mongolia (37.4, 95% UI 29.4-45.9 per 100,000), followed by North Korea (21.6, 95% UI 16.4-27.1 per 100,000), China, South Korea, and Japan (Figure 1B and Multimedia Appendix 2). The highest ASDR for GC was Mongolia (930.4, 95% UI 747.5-1157.9 per 100,000), followed by North Korea (585.7, 95% UI 437.3-748.5 per 100,000), China, South Korea, and Japan (Figure 1C and Multimedia Appendix 3). Taiwan (Province of China) had the lowest ASIR (11.5, 95% UI 10.2-12.7 per 100,000), ASMR (8.9, 95% UI 7.8-9.8 per 100,000), and ASDR (193.7, 95% UI 174.5-213.3 per 100,000) deserve mentioning.

**Figure 2.**
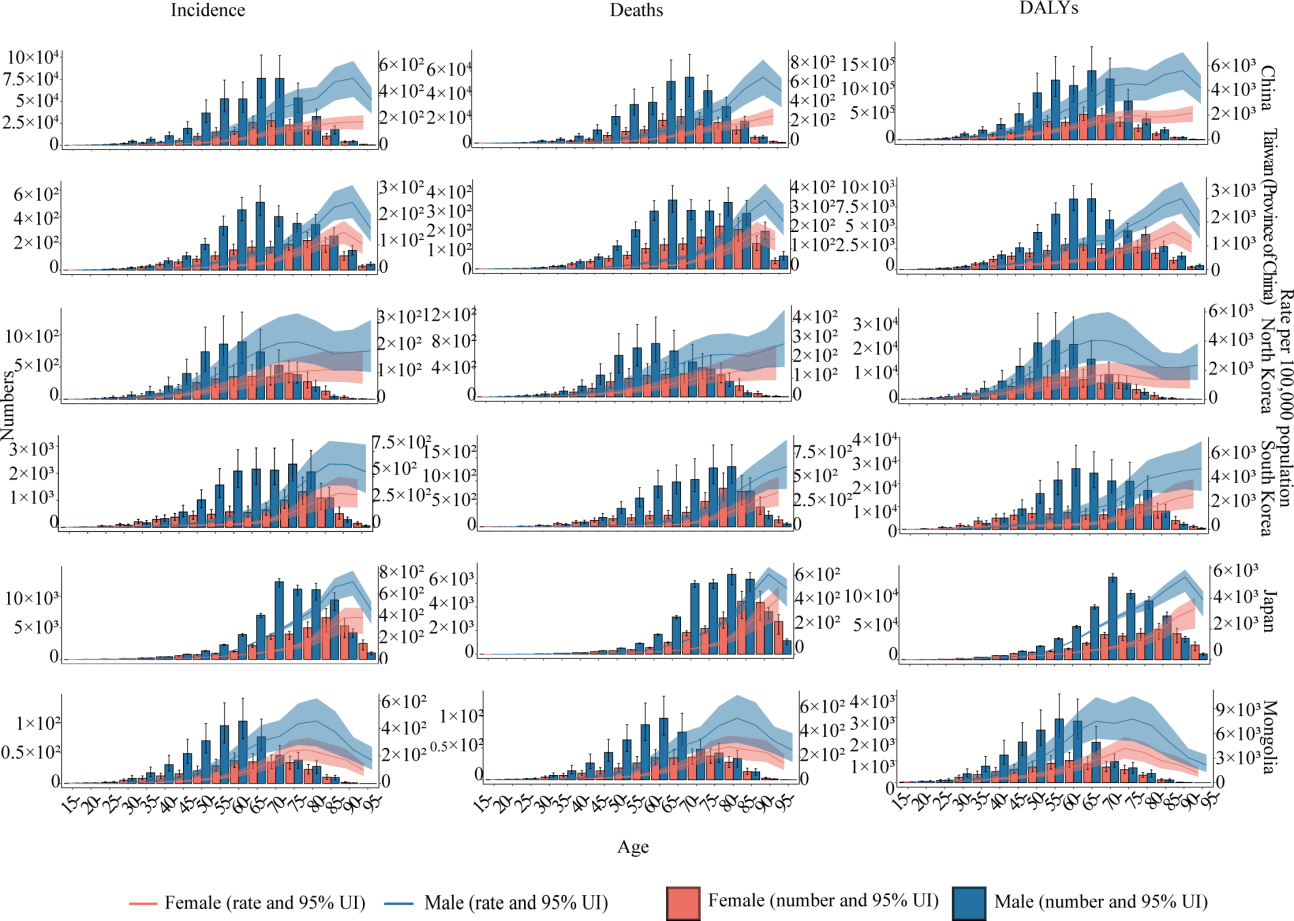
The age-specific burden of gastric cancer, incidence number and rate, deaths number and rate, and DALY number and rate in 2021. DALY: disability-adjusted life year; UI: uncertainty interval.

In Eastern Asia, the burden of GC was greater in the male population than the female population, including incidence, mortality, and DALYs. It is worth noting that the burden of GC was higher in the female population than the male population at age 90 years and older in Japan, 85 years and older in South Korea, and 75 years and older in North Korea. This phenomenon was caused by the different changes of incidence, mortality, and DALYs in the male and female population. For example, the incidence, mortality, and DALYs of GC rose with age among the female population, while the male population reached a peak and began to decline at 90‐94 years in Japan (Figure 2).

### Trend of GC Burden From 1990 to 2021

**Figure 3.**
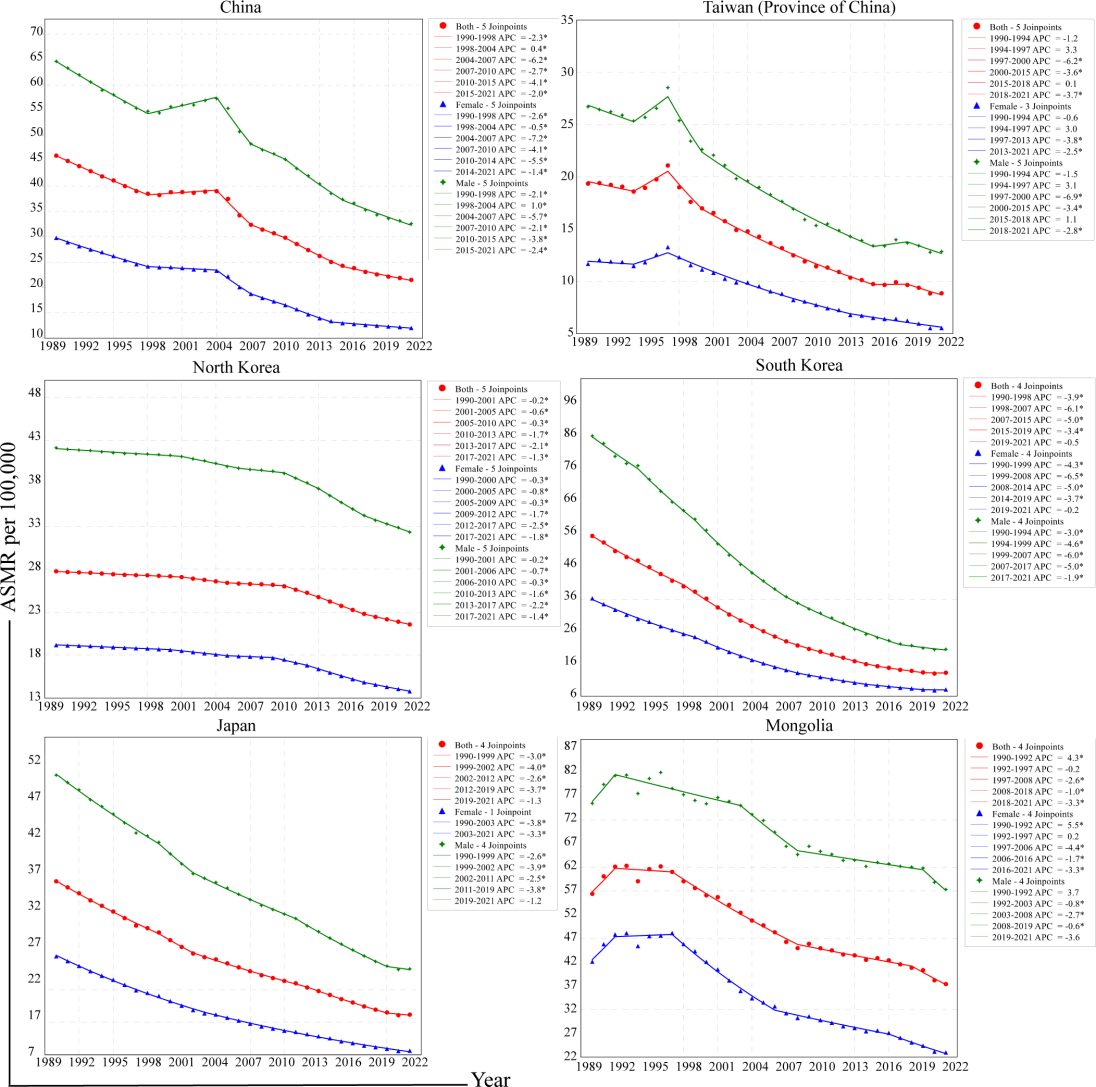
The age-standardized death trends of gastric cancer by sex in Eastern Asia, 1990-2021. APC: annual percent change; ASMR: age-standardized mortality rate.* indicates that 95% CI did not include 0.

The results of the joinpoint regression analysis indicated that GC incidence, mortality, and DALYs in Eastern Asia showed trends of decrease from 1990 to 2021, where similar trends were noted. However, there was significant heterogeneity in the temporal trends across countries (Table 1 and Figure 3). South Korea presented the highest absolute AAPC values for incidence, mortality, and DALYs, which represented that ASIRs, ASMRs, and ASDRs experienced the greatest declines in Eastern Asia. For example, the mortality of GC for both sexes dropped from 55.4 (95% UI 44.1-62.4) per 100,000 to 13.3 (95% UI 10.9-16.5) per 100,000 between 1990 and 2021 in South Korea, with the AAPC of **−**4.5 (95% CI −4.8 to −4.3). Notably, South Korea experienced the most rapid decline in GC mortality between 1998 and 2007, with the APC of −6.1 (95% CI −6.3 to −5.9). However, this trend decelerated subsequently, resulting in the APC of −0.5 (95% CI −3.2 to 2.4) between 2019 and 2021. In Japan, ASIR, ASMR, and ASDR also showed downward trends, but the absolute AAPC was smaller than that of South Korea, and the AAPC of ASMR was **−**3.0 (95% CI −3.2 to −2.8). From 1990 to 2021, ASIR, ASMR, and ASDR in China presented decreasing trends, and the AAPC of ASMR was **−**2.4 (95% CI −2.6 to −2.3). During the period from 1998 to 2004, this trend maintained stable or increased slightly, followed by the most rapid decline between 2004 and 2007, with the APC of −6.2 (95% CI −7.3 to −5.1), and the APC was −2.0 (95% CI −2.3 to −1.7) between 2015 and 2021.

The overall trend of ASIR, ASMR, and ASDR in North Korea remained stable between 1990 and 2021, AAPC of ASMR was **−**0.8 (95% CI −0.8 to −0.8). The mortality of GC for both sexes changed from 27.8 (95% UI 20.5-35.6) per 100,000 to 21.6 (95% UI 16.4-27.1) per 100,000 between 1990 and 2021 in North Korea, increased rank across countries. ASIR, ASMR, and ASDR of GC in Mongolia increased significantly from 1990 to 1992, then showed stability between 1992 and 1997 and declined slightly from 1997. In Mongolia, the ASMR of GC showed an overall slight decreasing trend for both sexes combined from 1990 to 2021 (AAPC **−**1.4, 95% CI −1.7 to −1.0), and Mongolia had the highest burden of GC among Eastern Asia in 2021. The ASIR, ASMR, and ASDR of GC in Taiwan (Province of China) were the lowest between 1990 and 2021. Although ASMR showed slight upward trends between 1994 and 1997, the overall trends were downward.

**Table 1.** Average annual percent change (AAPC) in incidence, mortality, and disability-adjusted life year (DALY) rates by sex in Eastern Asia, 1990‐2021.

Location and sex		China, percent change (95% CI)	Taiwan (Province of China), percent change (95% CI)	North Korea, percent change (95% CI)	South Korea, percent change (95% CI)	Japan, percent change (95% CI)	Mongolia, percent change (95% CI)
AAPC of incidence rate							
	Both	−1.6 (−1.7 to −1.5)	−2.0 (−2.7 to −1.3)	−0.5 (−0.5 to −0.4)	−3.2 (−3.5 to −3.0)	−3.0 (−3.2 to −2.7)	−1.3 (−1.6 to −0.9)
	Male	−1.3 (−1.5 to −1.2)	−1.7 (−2.0 to 1.4)	−0.5 (−0.6 to −0.5)	−3.3 (−3.6 to −3.1)	−2.9 (−3.1 to −2.7)	−0.8 (−1.3 to −0.3)
	Female	−2.2 (−2.3 to −2.0)	−2.0 (−2.6 to −1.3)	−0.8 (−0.8 to −0.7)	−3.4 (−3.7 to −3.2)	−3.3 (−3.6 to −3.0)	−2.0 (−2.4 to −1.6)
AAPC of mortality rate							
	Both	−2.4 (−2.6 to −2.3)	−2.6 (−3.3 to −1.9)	−0.8 (−0.8 to −0.8)	−4.5 (−4.8 to −4.3)	−3.0 (−3.2 to −2.8)	−1.4 (−1.7 to −1.0)
	Male	−2.9 (−3.0 to −2.7)	−2.4 (−2.8 to −2.0)	−1.1 (−1.1 to −1.0)	−4.7 (−4.9 to −4.5)	−3.5 (−3.5 to −3.4)	−2.0 (−2.4 to −1.6)
	Female	−2.2 (−2.4 to −2.0)	−2.4 (−3.2 to −1.5)	−0.8 (−0.9 to −0.8)	−4.6 (−4.8 to −4.3)	−2.9 (−3.1 to −2.7)	−0.9 (−1.4 to −0.4)
AAPC of DALY rate							
	Both	−2.8 (−2.9 to −2.6)	−2.9 (−3.6 to −2.2)	−0.7 (−0.8 to −0.7)	−5.1 (−5.3 to −4.8)	−3.4 (−3.6 to −3.2)	−1.5 (−1.8 to −1.2)
	Male	−2.5 (−2.7 to −2.3)	−2.7 (−3.5 to −1.9)	−0.8 (−0.8 to −0.7)	−5.1 (−5.4 to −4.8)	−3.3 (−3.5 to −3.1)	−0.9 (−1.5 to −0.3)
	Female	−3.2 (−3.4 to −3.1)	−2.8 (−3.0 to −2.6)	−1.0 (−1.1 to −1.0)	−5.2 (−5.4 to −4.9)	−3.8 (−4.1 to −3.5)	−2.3 (−2.7 to −1.9)

### Age-Specific Mortality Rates for GC

We arranged the mortality and population data into consecutive 5-year periods from 1992‐1996 (median 1994, IQR 1993-1995) to 2017‐2021 (median 2019, IQR 2018-2020) and 22 consecutive birth cohorts, including those born from 1895‐1899 (median 1897, IQR 1896-1898) to 2000‐2004 (median 2002, IQR 2001-2003). The results showed a declining trend in GC mortality among Eastern Asia during the period from 1992‐1996 to 2017‐2021.

**Figure 4.**
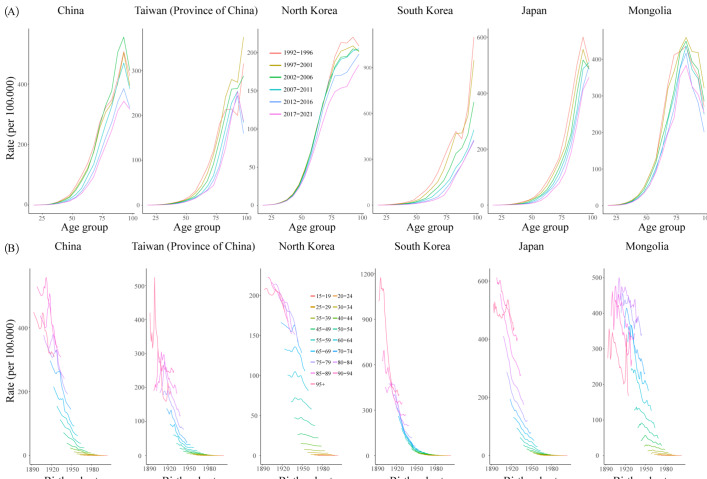
Age-specific mortality rates of gastric cancer (GC) by period and cohort-specific mortality rate of GC by age group in Eastern Asia, 1992‐2021. (A) Survey years were arranged into consecutive 5-year periods and describe the relationship between GC mortality rates and age group. (B) The GC mortality rates were arranged into 22 consecutive birth cohorts and describe the relationship between GC mortality rates and birth cohorts.

The mortality rate of GC in South Korea, North Korea, and Japan basically increased with the age group, while in China, Taiwan (Province of China), and Mongolia, the mortality rate of GC reached the peak around the age of 90 years, then decreased (Figure 4A). Eastern Asia showed a generally decreasing trend of GC mortality across birth cohorts, but old age groups in some countries showed an increase and then a decrease, indicating a relatively lower risk of GC mortality in cohorts born more recently (Figure 4B). For example, the mortality rate of GC in China decreased in 60‐64 years of age (median 62 years of age, IQR 61-63) across the periods of 1994, 1999, 2004, 2009, 2014, and 2019, with 138.0, 122.4, 116.4, 96.8, 74.6, and 64.4 per 100,000. In successive birth cohorts, the GC mortality rate gradually decreased in 60‐64 years of age (median 62 years of age, IQR 61-63).

### Net Drift and Local Drift in Different Age Groups

Net drift refers to the overall APC across the whole study period, while local drift refers to the APCs in the mortality rate for each age group relative to net drift (Table 2). The results of net drift showed that GC mortality had a downward trend in Eastern Asia, with the greatest reduction in South Korea and the smallest in North Korea. In China and Mongolia, mortality of the female population decreased more than the male population; nevertheless, there were fewer substantial reductions in mortality among the female population than the male population in South Korea. Local drift showed that the most significant decrease in mortality was in the 25‐ to 29-year age group for the male population in Korea (−7.3%, 95% CI −9.2 to −5.4), with the decline slowing as age groups increased. For the female population, the most pronounced decline seen was in the 65‐ to 69-year age group (−7.1%, 95% CI −7.5 to −6.8). Among the female population in Mongolia, the mortality rate decreased more in age groups younger than 75 years, but the mortality rate of the male population showed little decline in all age groups.

**Table 2.** Local drift with net drift values for gastric cancer in Eastern Asia, 1990‐2021.

Age group	China		Taiwan (Province of China)		North Korea		South Korea		Japan		Mongolia	
	Male	Female	Male	Female	Male	Female	Male	Female	Male	Female	Male	Female
Net drift	−2.6^a^	−3.7^a^	−3.1^a^	−3.2^a^	−0.9^a^	−1.1^a^	−5.9^a^	−5.5^a^	−3.8^a^	−4.0^a^	−1.1^a^	−3.0^a^
17.5	−3.1	−4^a^	−2.8	−2.6	−1.0	−1.6	−6.6	−6.3	−4.2	−4.0	−2.1	−2.8
22.5	−2.3^a^	−3.4^a^	−3.9	−4.2	−0.7	−1.3	−6.9^a^	−6.9^a^	−3.3^a^	−4.5^a^	−2.1	−3.6
27.5	−2.4^a^	−3.5^a^	−4.5^a^	−4.5^a^	−0.7	−1.0	−7.3^a^	−6.8^a^	−3.3^a^	−4.3^a^	−2.1	−4.0^a^
32.5	−2.6^a^	−3.7^a^	−4.3^a^	−4.1^a^	−0.8	−1.0	−7.3^a^	−5.7^a^	−3.8^a^	−4.5^a^	−1.8	−4.0^a^
37.5	−3.1^a^	−4.1^a^	−3.9^a^	−3.7^a^	−0.8^a^	−1.0^a^	−6.9^a^	−4.6^a^	−4.5^a^	−4.8^a^	−1.3	−3.7^a^
42.5	−3.4^a^	−4.4^a^	−3.3^a^	−3.4^a^	−0.8^a^	−1.0^a^	−6.6^a^	−4.3^a^	−5.3^a^	−5.3^a^	−1.0	−3.8^a^
47.5	−3.6^a^	−4.7^a^	−2.6^a^	−2.7^a^	−0.8^a^	−1.1^a^	−6.0^a^	−4.4^a^	−5.4^a^	−5.1^a^	−0.7	−4.1^a^
52.5	−3.4^a^	−4.6^a^	−2.6^a^	−2.9^a^	−0.9^a^	−1.1^a^	−6.1^a^	−5.4^a^	−4.9^a^	−4.6^a^	−0.5	−4.3^a^
57.5	−3.3^a^	−4.6^a^	−2.9^a^	−3.3^a^	−0.8^a^	−1.1^a^	−6.3^a^	−6.3^a^	−4.3^a^	−4.1^a^	−0.4	−4.2^a^
62.5	−2.9^a^	−4.2^a^	−3.5^a^	−3.7^a^	−0.8^a^	−1.1^a^	−6.3^a^	−7.1^a^	−3.7^a^	−3.6^a^	−0.7	−3.9^a^
67.5	−2.6^a^	−3.9^a^	−3.8^a^	−4^a^	−0.8^a^	−1.1^a^	−6.2^a^	−7.1^a^	−3.3^a^	−3.5^a^	−1.1^a^	−3.1^a^
72.5	−2.2^a^	−3.4^a^	−3.7^a^	−3.8^a^	−0.8^a^	−1.1^a^	−5.8^a^	−6.6^a^	−3.1^a^	−3.7^a^	−1.5^a^	−2.4^a^
77.5	−1.9^a^	−2.9^a^	−3^a^	−3.2^a^	−0.9^a^	−1.2^a^	−4.9^a^	−5.6^a^	−2.9^a^	−3.6^a^	−1.3^a^	−1.4^a^
82.5	−1.3^a^	−2.3^a^	−2.1^a^	−2.4^a^	−1.0^a^	−1.1^a^	−4.2^a^	−4.6^a^	−2.5^a^	−3.2^a^	−1.0	−0.8
87.5	−0.9^a^	−1.9^a^	−1.1^a^	−1.4^a^	−1.1	−1.1^a^	−3.3^a^	−3.4^a^	−2.1^a^	−2.6^a^	−1.1	−0.2
92.5	−0.7	−1.7^a^	−1	−0.8	−1.1	−0.9	−3.1^a^	−3.0^a^	−1.7^a^	−1.6^a^	−1.4	0.2
97.5	0	−1.7	−2.3	−1.2	−0.9	−0.6	−3.9^a^	−3.6^a^	−1.0^a^	−0.4	−1.3	0.7

a 95% CI did not include 0.

### Age-Period-Cohort Effects on GC Mortality

Multimedia Appendix 4 shows the effect of age, period, and cohort on GC in Eastern Asia. With advancing age, the RRs of GC mortality increased in Eastern Asia, while the mortality rate of GC in Korea increased slowly with age. In Mongolia, the highest mortality rate was in the 80‐ to 84-year age group, followed by a significant downward trend. Period and cohort effects in Eastern Asia tended to show similar trends. In Eastern Asia, the risk of GC mortality decreased compared with the control period set, indicating improvement in the health status of the whole population during the study period. The greatest improvement was in South Korea. In all birth cohorts, the most significant improvement compared to the reference cohort occurred among those born in South Korea before 1950. For the older cohorts, Taiwan (Province of China), China, and Japan all showed favorable improvements, while the improvement trends in North Korea and Mongolia were not evident. Among those born after 1950, there was an improvement trend in Eastern Asia, also with South Korea showing the most significant improvement, followed by Japan and China.

### DALYs From GC Attributable to Risk Factors

The percentage of age-standardized DALYs attributable to high-sodium diets and smoking in Eastern Asia is shown in Multimedia Appendix 5. There was no significant change in the population attributable scores for the high-sodium diets factor in 2021 compared to 1990. The ASDRs attributed to smoking among the female population in Eastern Asia were decreasing, and the proportion was not high, all being less than 5%. For the male population, the ASDRs attributed to smoking increased in Mongolia, showed no significant change in China, and showed downward trends in Japan, South Korea, North Korea, and Taiwan (Province of China). Among them, Japan declined the most, dropping from 22.4% (95% UI 18.4-26.8) in 1990 to 13.3% (95% UI 10.9-15.9) in 2021. In 2021, the ASDRs attributed to smoking were the highest at 19.1% (95% UI 16.1-22.2) for the male population in China.

**Figure 5.**
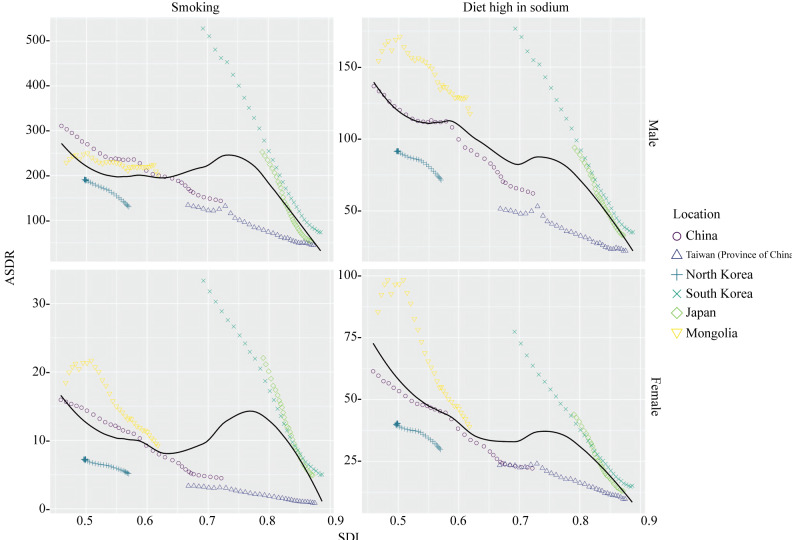
ASDR per 100,000 population for gastric cancer attributable to each risk factor for Eastern Asia by SDI, 1990‐2021. Expected values based on SDI and ASDR in all locations are shown as the black line. For each region, points from left to right depict estimates from each year from 1990 to 2021. ASDR: age-standardized disability-adjusted life year rate; SDI: sociodemographic index.

In Eastern Asia, the ASDRs for GC attributed to both factors showed overall downward trends with increasing SDI (Figure 5). As SDI increased year on year, from middle-high SDI in 1990 to high SDI in 2021, the ASDRs for both factors in Japan and South Korea decreased most rapidly. In Taiwan (Province of China), the rates of decline were slower because the ASDRs for both factors were already at relatively low levels in 1990. Between 1990 and 2021, as China moved from low-middle SDI to middle-high SDI, the ASDR for smoking and high-sodium diets declined relatively rapidly. In Mongolia, from 1990 to 2021, as the SDI transitioned from low-middle to middle level, the impact of smoking on male ASDR showed no significant improvement trend, while other factors demonstrated more noticeable trends of improvement. In North Korea, although the SDI increased between 1990 and 2021, it remained within the low-middle SDI level, and the reductions in both factors were relatively small.

### Projections of GC Mortality Trends

The projections of GC ASMR trends for the male and female population in Eastern Asia from 2022 to 2035 are shown in Multimedia Appendix 6. The results showed that the ASMR of GC for both male and female populations in Eastern Asia will continue to decrease, with the ASMR remaining higher in the male population than in the female population by 2035. Because the baseline is low, the decrease in GC mortality in Japan will be modest, with the ASMR for the female population decreasing from 7.7 (95% CI 7.6-7.8) per 100,000 in 2021 to 4.2 (95% CI 3.7-4.8) per 100,000 in 2035. In Japan, the rate of GC mortality in the male population is projected to decrease from 20.3 (95% CI 20.1-20.5) per 100,000 in 2021 to 11.1 (95% CI 10.1-12.1) per 100,000 in 2035, showing a greater decrease than that observed in the female population.

In China, GC mortality was projected to drop significantly, particularly for the female population, from 12.1 (95% CI 12.1-12.2) per 100,000 in 2021 to 6.9 (95% CI 5.5-8.3) per 100,000 by 2035. Nevertheless, GC mortality in Mongolia was expected to decrease slowly. The mortality rate of GC among the Mongolian female population will decrease from 22.6 (95% CI 20.9-24.2) per 100,000 in 2021 to 13.6 (95% CI 10.1-17.1) per 100,000 in 2035. In the male population among Mongolia, the ASMR will decrease from 59.4 (95% CI 56.8-62.0) in 2021 to 51.6 (95% CI 46.1-57.1) in 2035. It is predicted that the burden of GC among the Mongolian male population will remain the highest in Eastern Asia by 2035.

## Discussion

### Principal Findings

GC has long been a significant global health concern, with Eastern Asia standing out as a critical area for investigation due to its disproportionately high disease burden and distinct epidemiological characteristics. Comprising multiple countries, Eastern Asia shares certain cultural and dietary habits as well as some common genetic background elements. These intertwined factors play a vital role in the incidence and mortality of GC. Investigating the incidence, mortality, and DALYs due to GC in Eastern Asia is essential for dissecting the variation in epidemiological trends and underlying mechanisms among different countries and population subgroups. It not only provides evidence for public health decision makers to allocate resources more rationally but also formulates more effective strategies in prevention, screening, and treatment. In this study, we analyzed trends in mortality and ASMR of GC across Eastern Asia from 1990 to 2021 using data from the GBD and predicted the disease burden in 2035 that aimed to explore its spatiotemporal distribution patterns and identify potential intervention entry points, thereby laying a scientific theoretical foundation and practical guidance for reducing the burden of GC in this region.

In 2021, GC of Eastern Asia accounted for 53.2% (12,130,950/22,786,633) of total global DALYs, underscoring its substantial public health impact. Among the countries, Mongolia reported the highest ASIR, ASMR, and ADSR for GC, while Taiwan (Province of China) exhibited the lowest rates. These findings align with geographical distribution patterns in Eastern Asia previously noted in the 2019 GBD database [18]. GC presented a significantly greater risk to the male population compared to the female population in Eastern Asia, with the Chinese male population exhibiting the highest sex ratio of 2.4. This disparity was likely attributable to variations in lifestyle, environmental exposures, and genetic predispositions. The incidence, mortality, and DALYs of GC increased with age across both sexes, though the male population consistently exhibits higher rates—except in specific older age brackets, such as individuals older than 75 years in North Korea, older than 85 years in South Korea, and older than 90 years in Japan. These may be related to the protective effect of endogenous estrogen [19], so postmenopausal women were more at risk of GC. From 1990 to 2021, mortality rates for GC decreased across all of Eastern Asia. Analysis based on the AAPC revealed that declines in incidence and mortality were more pronounced in higher SDI countries, suggesting that economic development and health care improvements played pivotal roles in reducing GC disease burden.

However, the decline in incidence and mortality was not uniform across the countries. The steepest reduction was observed in South Korea, where mortality rates fell by 76% from 1990 to 2021. The local drift indicated the most significant decrease occurred within the 25‐ to 29-year age group among the male population in South Korea (−7.3%, 95% CI −9.2 to −5.4). Japan demonstrated the second-largest decline, with a 60.9% reduction. South Korea and Japan reflected successful experience in GC prevention and control. These findings highlight the potential for other countries to adopt similar strategies to achieve comparable outcomes. In China, GC mortality decreased by 53%, with the joinpoint analysis revealing a more significant decline from 2015 to 2021 (APC −2.0, 95% CI −2.3 to −1.7) compared to South Korea and Japan. Although mortality rates have declined, GC in North Korea has risen in ranking across Eastern Asia, and mortality rates reached 21.6 per 100,000 population. Meanwhile, Mongolia experienced a 33.7% decrease in mortality; yet, it continues to bear the world’s highest GC mortality rate at 38.2 per 100,000 population. This alarming statistic highlights the need for enhanced prevention strategies and early detection programs in Mongolia. In contrast, Taiwan has consistently maintained a lower burden, with a 54.2% reduction in GC mortality, reaching 8.86 per 100,000 population in 2021. The intrinsic estimation method was used to estimate the age, period, and cohort effects on Eastern Asia, which showed a generally decreasing trend of GC mortality in consecutive birth cohorts. Age-period-cohort analysis indicated a relatively lower risk of mortality from GC in individuals born in more recent cohorts. This pattern suggests that the decline in GC mortality is not only due to the improvement in medical treatment and early detection but also to the changes in lifestyle and dietary habits over the past few decades. The implementation of public health policies aimed at reducing the prevalence of *H. pylori* infection and the promotion of a balanced diet rich in fruits and vegetables may have contributed to this positive trend. Projections suggest a continued significant decrease in GC burden across Eastern Asia by 2035, although the Mongolian male population is expected to maintain the highest rates.

Our study showed that reductions in GC mortality across Eastern Asia have been driven by improved socioeconomic conditions, changes in key risk factors, and the introduction of cancer screening programs. The population-attributable scores for the high-sodium diet factor remained relatively unchanged in 2021 compared to 1990, maintaining a level of approximately 8% across all countries in Eastern Asia. However, the ASDR of GC from high-sodium diets decreased by 18.2% globally between 2007 and 2017 [20]. Therefore, it is pertinent to investigate the reasons underlying the lack of a downward trend in this risk factor across Eastern Asia over the past 3 decades. The WHO states that high-sodium diets raise the risk of GC [21]. It works by damaging the gastric mucosa with high salt concentrations, increasing susceptibility to *H. pylori* colonization and promoting gastric epithelial cell apoptosis in synergy with N-nitroso compounds and carcinogens [22]. A meta-analysis indicated that the risk of GC was increased by 12% per 5 g per day increment of dietary salt intake [23]. Traditional East Asian diets are high in salt when cooked or seasoned; despite countries having introduced policies to reduce salt intake, further efforts are needed. For example, sodium intake has decreased in South Korean people from 2012 but remains well above intake targets [24]. South Korean people consumed an average of 3.3 g of sodium daily in 2018, exceeding the WHO recommendation by over 1.5 times [25]. According to the data from the Korea National Health and Nutrition Examination Survey, the primary dietary sources of sodium intake among the South Korean people are salt, kimchi, and soy sauce, with daily kimchi consumption at 96.3 g [24]. In 2012, Japan formulated Health Japan 21 (the second term), with a target of consuming less than 8.0 g of salt per day [26]. However, the latest 2019 data showed that the average consumption was 9.7 g [27], well above the 5.0 g recommended by the WHO [21]. In Japan, sodium mainly comes from seasonings like soy sauce, table salt, and miso, as well as processed foods [26]. Sodium intake in China decreased from 6.3 g per day in 1991 to 4.1 g per day in 2015; yet, it remained twice the WHO’s recommended limit. In 2015, over 67% of sodium intake came from salt used in cooking [28]. In Eastern Asia, it is important to enhance nutrition education and develop low-sodium seasonings while improving food preservation methods to reduce sodium intake [29].

Smoking presented different attributable patterns for GC development. For the male population, the ASDRs attributed to smoking increased in Mongolia, showed no significant change in China and North Korea, and showed downward trends in Japan, South Korea, and Taiwan (Province of China). For the female population, smoking-related ASDRs decreased across Eastern Asia, even though the proportion was less than 5%. The Basic Plan to Promote Cancer Control programs, including tobacco control, was launched in 2017 in Japan [27]. Smoking prevalence among Japanese people has been decreasing gradually since 2004; it was 27.1% for the male population and 7.6% for the female population in 2019 [27]. Similarly, Taiwan (Province of China) implemented the Tobacco Hazards Prevention Act in 2009, reducing smoking prevalence in adults older than 40 years of age from 12.25% in 2001 to 8.18% in 2013 [30]. In contrast, after implementing various tobacco control measures, China reduced age-standardized smoking prevalence among men by 22.4%; it was still 37.5% in 2015 [31]. Smoking is an established cause of GC and was categorized as a class I carcinogen by the IARC [32]. Smoking significantly elevated GC risk, with meta-analyses indicating RRs of 1.30 (95% CI 1.23‐1.37) for current smokers and 1.53 (95% CI 1.44‐1.62) for former smokers compared to never-smokers [33]. The risk of GC intensified with increased smoking dosage and reached an RR of 1.69 for individuals smoking 20 cigarettes per day [33]. Another meta-analysis also confirmed this conclusion, with RRs for male and female population being 1.61 (95% CI 1.49-1.75) compared to those who never smoked [34]. Indeed, 16.5% of male and 1.9% of female patients with GC were estimated to be attributable to smoking globally in 2020 [35]. Consequently, it is imperative to enhance policy formulation and implementation to effectively achieve the objective of reducing smoking prevalence by 2030 [31].

As the proportion of DALYs attributed to high-sodium diet and smoking was not high, there were still risk factors outside the GBD data, such as *H. pylori* infection. The interaction between smoking and *H. pylori* infection further amplifies GC risk. For example, a 22.3-year follow-up study in Linqu County, a high-incidence area for GC in China, revealed that smoking significantly elevated the risk of GC incidence (odds ratio 1.72) and mortality (hazard ratio 2.01), but only among those infected with *H. pylori* [4]. This finding suggested a synergistic interplay between smoking and *H. pylori* infection in the pathogenesis of GC, underscoring the imperative for *H. pylori* eradication as a cornerstone of prevention, particularly in populations with high smoking prevalence. *H. pylori*, classified as a group I carcinogen by the IARC, remains a pivotal driver of GC. Notably, the cytotoxin-associated gene A positive strains of *H. pylori* in Eastern Asia are linked to a substantially greater risk of GC compared to Western populations [36]. Substantial evidence indicated that the eradication of *H. pylori* can reduce GC incidence by 46% (RR 0.54) in the general population and can also prevent progression in patients with mild to moderate atrophic gastritis [37,38]. The rs9607601 variant in the 22q13.1 region is associated with BAIAP2L2 expression, which is significantly overexpressed in GC tissues. BAIAP2L2 promotes the activation of the AKT/mTOR and Wnt/β-catenin pathways and may be involved in the development and progression of GC. Individuals carrying the rs9607601 T allele exhibit more pronounced therapeutic effects against *H. pylori* infection [39]. In 2015, the prevalence of *H. pylori* infection in Mongolia was strikingly high at 74.1%, surpassing rates in China (55.8%), Japan (51.7%), and South Korea (53.9%) [16]. Taiwan (Province of China) government launched an *H. pylori* eradication program and showed a strong association between the number of patients treated with *H. pylori* treatment and ASIR of GC (*r*=0.72) from 2005 to 2016 [30]. These results emphasize the need for targeted public health strategies in high-risk regions, focusing on smoking cessation, *H. pylori* screening and treatment, and sodium intake reduction to reduce the burden of GC.

Endoscopic screening represents a critical intervention for early detection and treatment of GC, with well-documented efficacy in high SDI countries such as Japan and South Korea. In Japan, nationwide GC screening began in the 1960s using radiography, evolving into a national program by 1983. Endoscopic screening was incorporated into the Japanese Guidelines for Gastric Cancer Screening in 2008, with biennial screening for individuals aged 50 years and older becoming the gold standard [40]. Hamashima et al [41] reported a significant reduction in mortality among the endoscopic screening cohort in Japan, with ASMR of 0.43 (95% CI 0.30-0.57). Similarly, in South Korea, the National Cancer Screening Program (NCSP), launched in 2001, offers biennial endoscopic or radiographic screening for individuals aged 40 years and older. NCSP started with free GC screening for Medical Aid recipients, then included lower-income NHI beneficiaries in 2005, and screenings are offered at 10% cost to the upper-income bracket thereafter. By providing low-cost or free screening options, the NCSP achieved high participation rates, contributing to a notable decline in GC mortality and an increase in the 5-year survival rate, which now surpasses that of Japan [40]. Jun et al [42] demonstrated that individuals who underwent endoscopic screening experienced a 21% lower risk of GC mortality compared to those who were not screened. Notably, frequent endoscopic examinations yielded a dose-dependent reduction in mortality, with odds ratios of 0.60, 0.32, and 0.19 for 1, 2, and 3 or more screenings, respectively [42]. The latest synthetic control study showed that the average RRs were 0.83 (95% CI 0.71-0.96) for GC mortality in South Korea. The RR reached 0.59 by the 15th year after the initiation of nationwide screening, while for Japan, the average RRs were 0.97 (95% CI 0.88-1.07) for GC mortality [43]. The benefits of South Korea’s countrywide GC screening were evident, but the success of Japan’s program was uncertain. Other countries in Eastern Asia could look to the experiences of South Korea and Japan as a reference.

It is worth mentioning that in China, since the inception of the National Key Public Health Projects in 2005, endoscopic screening programs have been implemented across more than 110 high-risk regions. Therefore, the mortality rate of GC in China increased slightly from 1998 to 2004 but had resumed the downward trend since 2005. China developed cancer screening guidelines of GC in 2022 on the basis of studies within the Chinese population [44]. Recent evidence from the National Cancer Center’s multicenter cohort study in China (2005‐2015) demonstrated that a single endoscopic screening significantly reduced the incidence and mortality of esophageal and noncardia GC as well as the mortality from cardia cancer, particularly among individuals aged 40‐69 years [45]. The local drift of this study showed that the mortality rate of the age group 40‐69 years decreased more than that of other age groups in China. Moreover, findings from Peking University Cancer Hospital’s research in Linqu County confirmed that endoscopic screening not only reduced the risk of invasive GC but also improved 5-year survival rates [46]. Repeated screenings further amplified these benefits, and the risk of GC death decreased more significantly in the repeat screening group than in the 1-time screening group. In addition, for individuals with a prior diagnosis of intestinal metaplasia or low-grade intraepithelial neoplasia, repetitive screening within 2 years can significantly improve the detection rate of early GC [46]. These findings underscore the importance of optimizing screening protocols, including the frequency and target population, to enhance early detection and treatment outcomes. Although China started GC screening later than South Korea and Japan, it has implemented effective screening programs and *H. pylori* prevention and treatment strategies and targeted attention to high-risk individuals. Therefore, the mortality of GC in China is expected to decrease significantly by 2035. However, although GC screening has been conducted for decades, it still faces some challenges. First, the coverage of GC screening remains relatively limited, and the compliance of target populations with screening is relatively low. Second, the quality of screening technologies varies significantly, and the process of screening, early diagnosis, and treatment is not sufficiently smooth.

Mongolia, where GC is the leading cause of cancer-related mortality, presents unique epidemiological challenges. The age of GC diagnosis is approximately a decade earlier than in other Eastern Asia countries, and projections indicate that Mongolia will remain the highest GC burden in Eastern Asia by 2035. Mongolia has a high prevalence of *H. pylori* infection significantly increasing the risk of GC [47]. In addition, dietary and lifestyle factors such as the consumption of excessively hot foods and beverages, a red meat-dominant diet, and frequent consumption of reheated leftovers may all exacerbate gastric carcinogenesis. Alarmingly, over 80% of GC cases in Mongolia are diagnosed at advanced stages, resulting in poor survival outcomes [48]. Addressing this pressing public health challenge will require a multifaceted approach, including public education campaigns to improve awareness about GC, increased access to affordable endoscopic screening, and enhanced health care infrastructure to facilitate timely diagnosis and treatment. The low level of socioeconomic development is a potential risk factor affecting the incidence and prognosis of GC [49]. As Mongolia is still considered to have a middle SDI, it is necessary to increase medical security to reduce the burden of GC. Encouragingly, the Mongolian government launched a nationwide noncommunicable disease screening program in 2019 [48], which holds promise for mitigating the rising burden of GC.

### Strengths and Limitations

Our study has some strengths, including the ability to visually display the risk of GC in Eastern Asia and its territories through risk area classification. This study has several limitations. First, the availability and quality of raw data restricted our ability to estimate the disease burden and the risk-attributed GC burden to a certain extent. Second, GCs exhibit heterogeneity in their pathogenesis, pathological characteristics, and epidemiological trends. We are unable to distinguish these subtypes for detailed statistical analysis.

### Conclusions

This analysis represents the first comprehensive evaluation of the GC burden in Eastern Asia using the latest GBD data. While ASIR, ASMR, and ASDR have declined over the past 3 decades, the absolute number of new cases, deaths, and DALYs attributable to GC has increased due to population growth and aging. These epidemiological trends highlight the enduring public health challenge posed by GC in Eastern Asia. To combat this burden effectively, a dual focus on primary and secondary prevention is paramount. Interventions should prioritize modifiable risk factors, including improved hygiene, dietary changes (eg, reduced sodium intake and increased consumption of fruits and vegetables), and the widespread adoption of *H. pylori* eradication strategies. Concurrently, enhanced screening programs and early diagnostic interventions must be implemented to achieve substantial reductions in mortality and improve survival outcomes. In the future, it is necessary to improve the detection rate of GC screening by accurately stratifying and identifying high-risk populations for GC. Artificial intelligence–assisted diagnostic technology and high-quality endoscopic examination are crucial for accurate diagnosis [50].

## Supplementary material

10.2196/75728 Multimedia Appendix 1 Incidence number and age-standardized incidence rate for stomach cancer in Asia, 1990 and 2021, and the estimated annual percentage change of 1990-2021, by regions.

10.2196/75728 Multimedia Appendix 2 Deaths number and age-standardized mortality rate for stomach cancer in Asia, 1990 and 2021, and the estimated annual percentage change of 1990-2021, by regions.

10.2196/75728 Multimedia Appendix 3 Disability-adjusted life year number and age-standardized disability-adjusted life year rate for stomach cancer in Asia, 1990 and 2021, and the estimated annual percentage change of 1990-2021, by regions.

10.2196/75728 Multimedia Appendix 4 The longitudinal age-specific mortality curve, period rate ratio, and cohort rate ratio for stomach cancer in East Asia. Reference period (2002-2007) and cohort (1947).

10.2196/75728 Multimedia Appendix 5 Percentage of gastric cancer age-standardized DALYs attributable to high-sodium diets and smoking by sex in Eastern Asia, 1990 and 2021. DALY: disability-adjusted life year.

10.2196/75728 Multimedia Appendix 6 The age-standardized mortality rate (ASMR) of gastric cancer was assessed in Eastern Asia from 1990 to 2021, with forecasted ASMR values projected for 2022 to 2035.

## Abbreviations

AAPC: average annual percent change
APC: annual percent change
ASDR: age-standardized disability-adjusted life year rate
ASIR: age-standardized incidence rate
ASMR: age-standardized mortality rate
BAPC: Bayesian age-period-cohort
DALY: disability-adjusted life year
GATHER: Guidelines for Accurate and Transparent Health Estimates Reporting
GC: gastric cancer
IARC: International Agency for Research on Cancer
*ICD*: *International Classification of Diseases*
NCSP: National Cancer Screening Program
PAF: Population Attributable Fraction
RR: relative risk
SDI: sociodemographic index
UI: uncertainty interval
WHO: World Health Organization
