# Supplementary material for: The Burden and Risk Factors of Gastric Cancer in Eastern Asia From 1990 to 2021: Longitudinal Observational Study of the Global Burden of Disease Study 2021
Source: JMIR Cancer. 2025 Aug 8;11:e75728. doi: 10.2196/75728 (PMC12334143; doi:10.2196/75728)
Supplement: Multimedia Appendix 1 [file cancer-v11-e75728-s001.docx]

| **Location** | **1990** | | **2021** | | **1990-2021** |
| --- | --- | --- | --- | --- | --- |
|  | **Incidence number (95% UI)** | **ASIR (95% UI)** | **Incidence number (95% UI)** | **ASIR (95% UI)** | **EAPC (95% CI)** |
| Afghanistan | 2758.9(1608.2 to 3775.4) | 39.7(23.3 to 53.7) | 3478.8(1874.1 to 4817.8) | 32.9(17.9 to 45) | -0.8(-1.0 to -0.6) |
| Armenia | 616.3(582.6 to 652.2) | 22.2(20.8 to 23.6) | 486.2(429.4 to 556.9) | 11.2(9.9 to 12.8) | -1.8(-2.0 to -1.6) |
| Azerbaijan | 1311.4(1073 to 1540.9) | 25.6(20.9 to 30.1) | 1460.8(1125.1 to 1974.4) | 14.4(11.3 to 19.2) | -1.8(-2.0 to -1.7) |
| Bahrain | 20.9(17.3 to 25) | 13.6(11.3 to 16.3) | 51.4(41.1 to 62.4) | 7(5.6 to 8.5) | -2.6(-2.9 to -2.3) |
| Bangladesh | 4805.2(3274.3 to 5927.3) | 9.9(6.8 to 12.2) | 7371.9(5007.4 to 9488) | 5.4(3.6 to 6.9) | -1.9(-2.1 to -1.7) |
| Bhutan | 20.9(13.5 to 29.4) | 8.1(5.4 to 11.2) | 33.3(23.3 to 45.2) | 5.5(3.9 to 7.5) | -1.2(-1.4 to -1.1) |
| Brunei Darussalam | 29.5(21.7 to 35.4) | 27.2(20 to 32.6) | 44.8(36.5 to 54.4) | 13(10.6 to 15.4) | -2.3(-2.5 to -2.0) |
| Cambodia | 748.8(561 to 950.2) | 16.1(12 to 20.5) | 1288.4(949.7 to 1685.1) | 10.5(8 to 13.5) | -1.5(-1.6 to -1.4) |
| China | 407471.3(337565.4 to 477568.6) | 48(40.2 to 56.7) | 611799(471965.8 to 765562.3) | 29.1(22.4 to 36.2) | -1.6(-1.8 to -1.5) |
| Cyprus | 82.3(69.2 to 110.1) | 11.8(9.9 to 15.8) | 136.1(105.5 to 163.9) | 6.8(5.2 to 8.1) | -1.3(-1.5 to -1.1) |
| Georgia | 1123.2(1026 to 1218.7) | 17.9(16.3 to 19.3) | 719.8(643.7 to 801) | 12.2(10.9 to 13.6) | -0.2(-0.7 to 0.2) |
| India | 38091.5(32483.9 to 49779.1) | 7.8(6.6 to 10.3) | 70163.4(61019 to 86919.3) | 5.8(5.1 to 7.2) | -0.8(-0.9 to -0.7) |
| Indonesia | 9454.8(7341.8 to 11664.8) | 9.3(7.1 to 11.7) | 17500.2(13968.4 to 22634.6) | 7.5(6 to 9.8) | -0.7(-0.7 to -0.6) |
| Iran (Islamic Republic of) | 5741.1(4088 to 6402.5) | 23.1(16.4 to 25.8) | 10381(6995.2 to 11530.9) | 13.9(9.3 to 15.4) | -1.5(-1.6 to -1.3) |
| Iraq | 542.6(430.7 to 731.8) | 6.6(5.2 to 8.9) | 1288.2(943.6 to 1687.8) | 5.4(4 to 6.9) | -0.9(-1.0 to -0.7) |
| Israel | 548.3(506.3 to 587.2) | 11.4(10.5 to 12.2) | 745.7(648.3 to 824.3) | 6(5.2 to 6.6) | -2.5(-2.7 to -2.3) |
| Japan | 108285.9(103063.9 to 111463.2) | 64(60.7 to 66) | 99035(85266.1 to 106694.5) | 25.5(23 to 27) | -3.0(-3.0 to -2.9) |
| Jordan | 98(79.2 to 122.4) | 7.2(5.9 to 9.1) | 293.9(222.3 to 380.8) | 4.1(3.1 to 5.2) | -1.9(-2.2 to -1.7) |
| Kazakhstan | 4358.6(4059.4 to 4685.3) | 33.8(31.4 to 36.5) | 2069.9(1786.8 to 2374.2) | 11.3(9.8 to 12.9) | -3.3(-3.5 to -3.2) |
| Kuwait | 26.4(23.7 to 29.8) | 4.3(3.8 to 4.9) | 84.7(67.6 to 105.6) | 3(2.4 to 3.8) | -1.3(-1.7 to -0.9) |
| Kyrgyz Republic | 961.5(871.5 to 1055.7) | 31.6(28.6 to 34.6) | 792(646.7 to 943.9) | 15.8(13 to 18.9) | -2.0(-2.2 to -1.8) |
| Lao People's Republic | 363(255.2 to 480.4) | 16.9(12 to 22.2) | 389.3(286.9 to 505.2) | 8.4(6.2 to 10.9) | -2.4(-2.5 to -2.4) |
| Lebanese Republic | 221.3(178 to 280.5) | 10.5(8.4 to 13.2) | 354.4(285.2 to 431.3) | 5.8(4.6 to 7) | -1.6(-1.8 to -1.4) |
| Malaysia | 807.5(677.7 to 955.1) | 8.7(7.3 to 10.3) | 1891.6(1613.8 to 2303.7) | 6.8(5.8 to 8.2) | -1.0(-1.1 to -0.8) |
| Maldives | 8.4(6.5 to 10.5) | 9.3(7.1 to 11.7) | 10.3(7.9 to 13.1) | 3(2.3 to 3.9) | -4.0(-4.2 to -3.8) |
| Mongolia | 573.8(466.5 to 714.5) | 54.2(44 to 67.9) | 848.3(680.3 to 1062.3) | 36.8(29.4 to 45.3) | -1.6(-1.7 to -1.4) |
| Myanmar | 3635(2645.7 to 4688.5) | 15.2(11.1 to 19.4) | 3568.4(2750.3 to 4705.2) | 7.4(5.7 to 9.7) | -2.7(-2.8 to -2.5) |
| Nepal | 838.2(584.6 to 1093.3) | 8.6(6.1 to 11) | 1407.9(1027.5 to 1868.6) | 6.1(4.5 to 8.1) | -1.0(-1.3 to -0.7) |
| North Korea | 4622.1(3365.1 to 6013.4) | 27.9(20.6 to 35.8) | 8049.8(6046.3 to 10116.4) | 24(18.2 to 30.2) | -0.4(-0.6 to -0.3) |
| Oman | 77.2(56.2 to 103.3) | 11.2(8.2 to 14.8) | 109.3(83.3 to 136.9) | 5.8(4.4 to 7.2) | -1.8(-1.9 to -1.6) |
| Pakistan | 2957(2392.8 to 3718.3) | 5.2(4.3 to 6.6) | 5827(4556.9 to 7473.4) | 4.7(3.7 to 6) | -0.7(-1.0 to -0.4) |
| Palestine | 95.3(73.4 to 121.6) | 11.5(8.9 to 14.5) | 151.2(119.4 to 180.4) | 6.4(5.1 to 7.6) | -2.0(-2.2 to -1.7) |
| Philippines | 1662.7(1427.7 to 1989.6) | 5.4(4.7 to 6.6) | 3734.3(3086.2 to 4990.7) | 4.5(3.7 to 5.9) | -0.5(-0.6 to -0.4) |
| Qatar | 15(11.6 to 18.9) | 15.8(12.3 to 19.5) | 54.3(39.7 to 73.9) | 6.5(4.9 to 8.4) | -3.1(-3.7 to -2.5) |
| Saudi Arabia | 363.7(258.3 to 552.2) | 6.3(4.5 to 9.2) | 795.8(600.7 to 1268) | 3.9(3.1 to 5.8) | -1.6(-1.8 to -1.5) |
| Singapore | 509.7(477.4 to 540.1) | 23.1(21.6 to 24.5) | 708(634.1 to 773.7) | 8.5(7.6 to 9.3) | -3.2(-3.4 to -3.0) |
| South Korea | 22166.6(16776.6 to 25106.9) | 71.2(56 to 80.7) | 23663.6(19710.1 to 29807.1) | 25.8(21.5 to 32.4) | -3.5(-3.7 to -3.4) |
| Sri Lanka | 918.5(761.5 to 1067.2) | 8.6(7.1 to 10) | 1099(709.1 to 1491.3) | 4.1(2.6 to 5.5) | -2.5(-2.8 to -2.3) |
| Syrian Arab Republic | 405.4(312.3 to 509.1) | 7.8(6 to 9.9) | 774.4(570.1 to 1034.6) | 6.3(4.7 to 8.1) | -0.9(-1.0 to -0.8) |
| Taiwan (Province of China) | 3427.4(2983 to 3756.2) | 21.5(18.7 to 23.6) | 4839.4(4272.3 to 5352.2) | 11.5(10.2 to 12.7) | -2.5(-2.8 to -2.2) |
| Tajikistan | 796.1(648.1 to 935.2) | 28.5(23.4 to 33.5) | 836.4(649.9 to 1093) | 14.2(11.2 to 18.5) | -2.2(-2.4 to -2.0) |
| Thailand | 4170.8(2930.4 to 5096) | 11.5(8 to 13.9) | 9165.4(5448.4 to 12170) | 8.7(5.2 to 11.5) | -1.3(-1.4 to -1.1) |
| Timor-Leste | 29.6(19.8 to 39.7) | 10.2(7 to 13.3) | 64.1(46.5 to 85.8) | 7.6(5.5 to 10.2) | -0.8(-1.1 to -0.6) |
| Turkey | 8422.5(6470 to 9894.5) | 24.1(18.7 to 28.3) | 11569.7(8585 to 14294.5) | 12.5(9.3 to 15.4) | -2.2(-2.5 to -1.8) |
| Turkmenistan | 426.2(392.9 to 460.8) | 21.7(19.9 to 23.5) | 408.7(310 to 535.5) | 9.8(7.4 to 12.7) | -2.6(-2.9 to -2.3) |
| United Arab Emirates | 64.1(47.4 to 85.2) | 14.4(10.8 to 18.7) | 229.7(175.5 to 314.6) | 7.8(6.1 to 10.1) | -0.5(-1.1 to 0.0) |
| Uzbekistan | 2427.3(2202.6 to 2647.1) | 20.7(18.7 to 22.7) | 2141.1(1723.4 to 2630.1) | 7.8(6.3 to 9.6) | -2.8(-3.0 to -2.6) |
| Viet Nam | 6061.6(4537.1 to 7706.2) | 14.9(11.2 to 18.9) | 8776.6(6719.7 to 11482.9) | 8.6(6.8 to 11.3) | -2.2(-2.4 to -1.9) |
| Yemen | 1180.5(620 to 1622.3) | 23.7(12.8 to 32) | 2611.3(1154.9 to 3686) | 18.7(8.4 to 26) | -1.0(-1.0 to -0.9) |

ASIR: Age standardized incidence rate. EAPC: Estimated annual percentage change. UI: Uncertain interval. CI: Confidence interval
