# Supplementary material for: The Burden and Risk Factors of Gastric Cancer in Eastern Asia From 1990 to 2021: Longitudinal Observational Study of the Global Burden of Disease Study 2021
Source: JMIR Cancer. 2025 Aug 8;11:e75728. doi: 10.2196/75728 (PMC12334143; doi:10.2196/75728)
Supplement: Multimedia Appendix 2 [file cancer-v11-e75728-s002.docx]

| **Location** | **1990** | | **2021** | | **1990-2021** |
| --- | --- | --- | --- | --- | --- |
|  | **Deaths number (95% UI)** | **ASMR (95% UI)** | **Deaths number (95% UI)** | **ASMR (95% UI)** | **EAPC (95% CI)** |
| Afghanistan | 2819.6(1682.6 to 3849.6) | 41.5(24.9 to 56) | 3468.7(1888.2 to 4779.6) | 34.6(19 to 46.9) | -0.8(-1.0 to -0.5) |
| Armenia | 594.7(559.5 to 630.9) | 21.8(20.4 to 23.3) | 469.4(411.3 to 539.4) | 10.8(9.4 to 12.4) | -1.9(-2.1 to -1.6) |
| Azerbaijan | 1293.7(1057.1 to 1524.8) | 25.9(21.1 to 30.4) | 1403.6(1082.3 to 1897.6) | 14.3(11.2 to 19.2) | -1.9(-2.0 to -1.7) |
| Bahrain | 20.1(16.7 to 24) | 14.2(11.8 to 16.9) | 43.7(34.9 to 53.4) | 6.8(5.4 to 8.3) | -2.8(-3.2 to -2.5) |
| Bangladesh | 4799.6(3287.7 to 5908.2) | 10.1(6.9 to 12.5) | 7242.7(4954.2 to 9344.2) | 5.4(3.7 to 7) | -2.0(-2.2 to -1.8) |
| Bhutan | 20.6(13.5 to 28.6) | 8.3(5.6 to 11.5) | 33.3(23.3 to 44.9) | 5.6(4 to 7.6) | -1.2(-1.4 to -1.1) |
| Brunei Darussalam | 23.9(17.5 to 28.7) | 23.6(17.4 to 28.2) | 32(26.2 to 38.4) | 10.1(8.3 to 12.1) | -2.5(-2.8 to -2.2) |
| Cambodia | 746.9(559.9 to 943.5) | 16.7(12.5 to 21.2) | 1228.8(915 to 1581.8) | 10.5(8.1 to 13.4) | -1.6(-1.7 to -1.5) |
| China | 374066.2(310921.1 to 442250.7) | 46(38.9 to 54.4) | 445012.7(344736.2 to 555834) | 21.5(16.7 to 26.6) | -2.5(-2.8 to -2.3) |
| Cyprus | 78.9(66.1 to 106.5) | 12(10 to 16.2) | 115.5(89.6 to 139.5) | 5.9(4.6 to 7.1) | -1.9(-2.1 to -1.7) |
| Georgia | 1085.5(994.1 to 1176.2) | 17.4(16 to 18.8) | 712.4(634.4 to 794.8) | 11.9(10.6 to 13.3) | -0.2(-0.7 to 0.3) |
| India | 37351.5(31720.8 to 48997.1) | 7.9(6.7 to 10.6) | 68517.1(59454.9 to 84301.8) | 5.8(5.1 to 7.2) | -0.8(-1.0 to -0.7) |
| Indonesia | 9277.7(7221.1 to 11513.3) | 9.6(7.3 to 12.1) | 16481(13146.7 to 21387.1) | 7.5(6 to 9.8) | -0.8(-0.8 to -0.7) |
| Iran (Islamic Republic of) | 5608.6(3993.1 to 6255.3) | 24(16.9 to 26.8) | 9576.1(6554 to 10537.5) | 13.3(9 to 14.6) | -1.7(-1.9 to -1.5) |
| Iraq | 533.6(422.7 to 717.1) | 6.6(5.2 to 8.9) | 1186.4(874.1 to 1515.9) | 5.3(3.9 to 6.7) | -1.0(-1.1 to -0.9) |
| Israel | 521.7(480.5 to 559) | 11(10.1 to 11.8) | 661.3(569.4 to 733.9) | 5.1(4.5 to 5.7) | -2.9(-3.1 to -2.7) |
| Japan | 56091.1(52923.3 to 57892.6) | 33.8(31.6 to 34.9) | 58011.8(48988.5 to 63019.3) | 13.2(11.7 to 14) | -3.1(-3.1 to -3.0) |
| Jordan | 94.7(76.6 to 118.1) | 7.4(6 to 9.3) | 260.2(199.4 to 335.9) | 3.9(3 to 4.9) | -2.2(-2.4 to -2.0) |
| Kazakhstan | 4262.8(3969 to 4599.5) | 33.6(31.2 to 36.3) | 1933.1(1662.6 to 2219.2) | 10.8(9.3 to 12.3) | -3.5(-3.7 to -3.3) |
| Kuwait | 23.5(21.3 to 26.4) | 4.2(3.7 to 4.7) | 66.8(54.6 to 82.8) | 2.6(2.1 to 3.2) | -1.7(-2.1 to -1.2) |
| Kyrgyz Republic | 941.9(853.7 to 1033.7) | 31.4(28.4 to 34.3) | 745.2(606.9 to 892.3) | 15.3(12.5 to 18.2) | -2.0(-2.3 to -1.8) |
| Lao People's Republic | 363.5(256.2 to 482.1) | 17.5(12.5 to 23) | 376.2(273.8 to 487.7) | 8.5(6.3 to 11) | -2.5(-2.6 to -2.4) |
| Lebanese Republic | 219.2(176.9 to 277.8) | 10.7(8.7 to 13.5) | 337.6(271.1 to 408.2) | 5.4(4.3 to 6.5) | -1.9(-2.1 to -1.8) |
| Malaysia | 797.8(666.7 to 934.1) | 8.9(7.4 to 10.4) | 1702.7(1458.2 to 2053.6) | 6.3(5.4 to 7.5) | -1.3(-1.4 to -1.1) |
| Maldives | 8.2(6.3 to 10.2) | 9.5(7.3 to 12) | 8.9(6.8 to 11.3) | 2.8(2.2 to 3.6) | -4.3(-4.6 to -4.1) |
| Mongolia | 585.5(476.4 to 734) | 56.4(45.8 to 71.2) | 824.8(659.4 to 1028.1) | 37.4(29.4 to 45.9) | -1.7(-1.8 to -1.5) |
| Myanmar | 3620.1(2638.9 to 4647.7) | 15.6(11.5 to 19.9) | 3443(2667.7 to 4540) | 7.4(5.8 to 9.8) | -2.8(-2.9 to -2.6) |
| Nepal | 831.3(583.1 to 1071.8) | 8.8(6.4 to 11.2) | 1393.3(1026.5 to 1839.7) | 6.2(4.6 to 8.2) | -1.0(-1.3 to -0.7) |
| North Korea | 4428(3194.9 to 5748.8) | 27.8(20.5 to 35.6) | 7135.2(5391.2 to 9040.5) | 21.6(16.4 to 27.1) | -0.8(-0.9 to -0.6) |
| Oman | 73.6(54.1 to 97.6) | 11.2(8.2 to 14.7) | 92.1(70.8 to 115) | 5.3(4.1 to 6.5) | -2.0(-2.1 to -1.8) |
| Pakistan | 3006.8(2442.8 to 3786.8) | 5.5(4.4 to 6.9) | 5737.9(4491.5 to 7323.3) | 4.9(3.8 to 6.1) | -0.7(-1.0 to -0.4) |
| Palestine | 97(75.3 to 123.5) | 12.1(9.5 to 15.2) | 141.5(114.1 to 168.2) | 6.4(5.1 to 7.6) | -2.1(-2.4 to -1.8) |
| Philippines | 1601(1374.2 to 1923.9) | 5.6(4.9 to 6.9) | 3498.1(2893.8 to 4610.4) | 4.4(3.6 to 5.7) | -0.6(-0.7 to -0.5) |
| Qatar | 13.6(10.5 to 17.2) | 16.1(12.7 to 19.8) | 39.8(29.6 to 53.9) | 5.8(4.6 to 7.5) | -3.6(-4.2 to -2.9) |
| Saudi Arabia | 359(258.1 to 536.5) | 6.5(4.8 to 9.5) | 663(504.9 to 1039.5) | 3.6(2.9 to 5.3) | -2.0(-2.2 to -1.8) |
| Singapore | 347.9(329 to 368) | 16.5(15.6 to 17.6) | 375(336.5 to 409.7) | 4.5(4.1 to 4.9) | -4.3(-4.5 to -4.0) |
| South Korea | 16260.7(12514.7 to 18306.1) | 55.4(44.1 to 62.4) | 12270.5(10052.3 to 15312.2) | 13.3(10.9 to 16.5) | -5.0(-5.2 to -4.9) |
| Sri Lanka | 895.8(742.4 to 1041.9) | 8.8(7.3 to 10.2) | 946.3(618.3 to 1284.9) | 3.6(2.4 to 4.8) | -3.0(-3.2 to -2.8) |
| Syrian Arab Republic | 402.8(310.4 to 503.7) | 8(6.2 to 10.1) | 715.9(534.1 to 940.6) | 6.1(4.7 to 7.8) | -1.1(-1.2 to -0.9) |
| Taiwan (Province of China) | 2940.6(2552.5 to 3219.4) | 19.3(16.8 to 21.1) | 3799(3344.8 to 4220.3) | 8.9(7.8 to 9.8) | -3.0(-3.2 to -2.7) |
| Tajikistan | 795.3(652.6 to 934.4) | 29(23.9 to 34.1) | 816.8(636.1 to 1070) | 14.6(11.5 to 18.9) | -2.1(-2.4 to -1.9) |
| Thailand | 3968(2791.5 to 4826.2) | 11.4(8 to 13.7) | 7748.5(4677.3 to 10227.1) | 7.3(4.4 to 9.6) | -1.8(-2.0 to -1.7) |
| Timor-Leste | 29(19.8 to 38.7) | 10.7(7.4 to 13.9) | 63.2(45.8 to 84.6) | 7.7(5.7 to 10.3) | -0.9(-1.2 to -0.7) |
| Turkey | 8309.6(6430.2 to 9757.4) | 24.6(19.3 to 28.8) | 10490.9(7916.6 to 12827.4) | 11.6(8.7 to 14.1) | -2.5(-2.8 to -2.1) |
| Turkmenistan | 421.1(387.2 to 455.8) | 22(20.1 to 23.8) | 390.4(299.9 to 510.3) | 9.6(7.4 to 12.5) | -2.7(-3.0 to -2.4) |
| United Arab Emirates | 60.4(44.8 to 80) | 14.8(11.2 to 19.1) | 196.1(151.6 to 263.8) | 8.1(6.4 to 10.3) | -0.4(-1.0 to 0.2) |
| Uzbekistan | 2385.1(2161.4 to 2606.8) | 20.6(18.6 to 22.6) | 2036.2(1634 to 2471.2) | 7.7(6.2 to 9.3) | -2.8(-3.0 to -2.6) |
| Viet Nam | 5982.8(4487.1 to 7555.3) | 14.9(11.3 to 18.8) | 7560.6(5902 to 9811.4) | 7.7(6.1 to 9.9) | -2.5(-2.8 to -2.3) |
| Yemen | 1175.7(631.7 to 1615.1) | 24.6(13.5 to 33.2) | 2582.9(1125.1 to 3662.2) | 19.4(8.7 to 27.1) | -1.0(-1.1 to -0.9) |

ASMR: Age standardized mortality rate. EAPC: Estimated annual percentage change. UI: Uncertain interval. CI: Confidence interval
