# Supplementary material for: The Burden and Risk Factors of Gastric Cancer in Eastern Asia From 1990 to 2021: Longitudinal Observational Study of the Global Burden of Disease Study 2021
Source: JMIR Cancer. 2025 Aug 8;11:e75728. doi: 10.2196/75728 (PMC12334143; doi:10.2196/75728)
Supplement: Multimedia Appendix 3 [file cancer-v11-e75728-s003.docx]

| **Location** | **1990** | | **2021** | | **1990-2021** |
| --- | --- | --- | --- | --- | --- |
|  | **DALYs number (95% UI)** | **ASDR (95% UI)** | **DALYs number (95% UI)** | **ASDR (95% UI)** | **EAPC (95% CI)** |
| Afghanistan | 82524.3(47147.7 to 114271.8) | 1137.8(659 to 1575.8) | 115055.3(60032.6 to 161225) | 910.2(498.9 to 1256.8) | -0.9(-1.1 to -0.7) |
| Armenia | 17234.1(16385.3 to 18116.9) | 588.6(556.8 to 621) | 11096.2(9812 to 12754.1) | 259.4(229.4 to 298) | -2.4(-2.6 to -2.2) |
| Azerbaijan | 38154.1(31197.7 to 44783.2) | 702.4(573.1 to 827.7) | 38529.6(29425.8 to 52566.8) | 350.1(267.5 to 474.8) | -2.4(-2.5 to -2.2) |
| Bahrain | 571.4(477.8 to 678.2) | 304.8(254.3 to 363.9) | 1229.7(979.5 to 1501.5) | 132.9(105.5 to 162) | -3.2(-3.5 to -2.9) |
| Bangladesh | 145099.6(97658.7 to 179537.1) | 275.3(186.6 to 339.9) | 191348(132083.7 to 248024.7) | 133(91.9 to 172.8) | -2.3(-2.4 to -2.1) |
| Bhutan | 656.3(429.4 to 917.6) | 225(147.6 to 313.6) | 851.1(579.7 to 1164.5) | 134.1(92.2 to 182.5) | -1.7(-1.8 to -1.6) |
| Brunei Darussalam | 712.2(527 to 865.9) | 568.7(417.2 to 683.9) | 927.7(751.4 to 1129.9) | 236.1(193.2 to 283.8) | -2.7(-3.0 to -2.4) |
| Cambodia | 22940.5(17346.5 to 28995.6) | 443.2(333.7 to 560.3) | 34560.6(25005.5 to 45438.4) | 257.4(188.6 to 334.7) | -1.9(-2.0 to -1.8) |
| China | 10773456.9(8850977.2 to 12638918.9) | 1181.6(978.4 to 1390.9) | 10642126.5(8222106.3 to 13383779.1) | 501.3(387.3 to 628) | -2.9(-3.1 to -2.7) |
| Cyprus | 1698.1(1425.9 to 2227.8) | 227.2(192.6 to 302.3) | 2239.9(1745.8 to 2729.1) | 111.9(86.2 to 135.1) | -1.9(-2.1 to -1.8) |
| Georgia | 30018.5(27355.2 to 32894.2) | 475.2(434.9 to 519.7) | 16683.7(14820 to 18638) | 294.3(261 to 329.1) | -0.6(-1.0 to -0.1) |
| India | 1176741.9(999342.7 to 1512841.8) | 217.8(185.2 to 284.1) | 1893705.6(1648775.3 to 2347624.7) | 149.3(129.8 to 184.7) | -1.1(-1.2 to -1.0) |
| Indonesia | 288529(225392.1 to 351637.2) | 253(196.2 to 313.5) | 463366.7(368891.9 to 589871.2) | 178.9(143.2 to 229.9) | -1.1(-1.2 to -1.0) |
| Iran (Islamic Republic of) | 158851.3(115664.3 to 176727) | 563.1(405.6 to 624.9) | 233777.9(164155.3 to 257563.7) | 292.6(202.3 to 322.1) | -1.9(-2.1 to -1.7) |
| Iraq | 15988.3(12570.1 to 21180.7) | 179.8(142 to 240.5) | 33948.9(24788.8 to 44402) | 126.7(93.1 to 162.3) | -1.4(-1.5 to -1.3) |
| Israel | 11116.4(10399 to 11878.1) | 233(217.5 to 249.3) | 12932(11497 to 14239.9) | 108.4(96.7 to 119.1) | -2.9(-3.0 to -2.7) |
| Japan | 1332552.2(1283575.4 to 1367533.2) | 790.8(759.6 to 811.8) | 925232.7(815855.4 to 984384) | 270.2(248.9 to 282.2) | -3.5(-3.5 to -3.4) |
| Jordan | 2884.6(2324.5 to 3594.4) | 184.7(149.4 to 230) | 7201(5505.9 to 9422.1) | 87.6(67.1 to 112.9) | -2.6(-2.9 to -2.4) |
| Kazakhstan | 123279.5(115266.2 to 131594.3) | 913.4(853.7 to 980.3) | 53831.6(46541.7 to 61807.3) | 280(242 to 321.1) | -3.7(-3.9 to -3.5) |
| Kuwait | 721.3(652.3 to 805.7) | 97.9(88 to 110.4) | 1818(1485.8 to 2261.6) | 54.9(44.3 to 68.5) | -2.0(-2.5 to -1.6) |
| Kyrgyz Republic | 28326.3(25574.1 to 31091) | 899.2(813.2 to 987.4) | 22055.8(18014.4 to 26270) | 407.9(333.5 to 486.7) | -2.3(-2.5 to -2.2) |
| Lao People's Republic | 11162.2(7821.5 to 14768.3) | 476.2(338.5 to 633) | 10941.9(7959.7 to 14429.5) | 211.2(154.3 to 275.6) | -2.8(-2.9 to -2.7) |
| Lebanese Republic | 5883.2(4740.5 to 7521.9) | 259.8(209.6 to 330.8) | 7133.4(5764.5 to 8649.9) | 118.7(95.9 to 144) | -2.3(-2.4 to -2.1) |
| Malaysia | 21957.9(18626 to 26191.9) | 219(184.8 to 259.3) | 43838.2(37604.4 to 53863.6) | 148.9(127.8 to 181.1) | -1.4(-1.5 to -1.2) |
| Maldives | 255.2(189.4 to 318.6) | 245.8(189.1 to 306.5) | 238.9(182.3 to 304.5) | 61.3(47.1 to 78) | -4.9(-5.2 to -4.6) |
| Mongolia | 16450(13255.4 to 20644.5) | 1462.9(1184.8 to 1839.7) | 24063.6(19036 to 30531.8) | 930.4(747.5 to 1157.9) | -1.8(-2.0 to -1.6) |
| Myanmar | 111607(79712.7 to 145417.4) | 425.7(310 to 549.4) | 94025.2(70953.1 to 124816) | 182.5(139.1 to 242.1) | -3.1(-3.3 to -3.0) |
| Nepal | 25849.6(17925.7 to 33537.9) | 238.5(166.7 to 308.1) | 37672(28087.9 to 50004.6) | 153.4(113.7 to 202.8) | -1.3(-1.6 to -1.0) |
| North Korea | 132582.5(94427.6 to 174136.8) | 739.7(531.9 to 961.9) | 200037.1(148083.1 to 255936) | 585.7(437.3 to 748.5) | -0.7(-0.9 to -0.6) |
| Oman | 2161.8(1571.1 to 2885.9) | 277(202.6 to 367.8) | 2642.1(2026.9 to 3334.1) | 115.7(88.8 to 143.9) | -2.5(-2.6 to -2.3) |
| Pakistan | 84312.4(68796.3 to 105988.6) | 138.6(112.8 to 175.6) | 172298.3(133303.7 to 220918.1) | 122(95.3 to 156.3) | -0.7(-1.1 to -0.4) |
| Palestine | 2451.1(1880.3 to 3154.7) | 272.4(209.9 to 347.8) | 3742.9(2985.3 to 4485.6) | 139.1(112.2 to 165.6) | -2.2(-2.4 to -2.0) |
| Philippines | 50710.9(43357.3 to 59383.4) | 143.9(123.3 to 172.2) | 101402.1(83388.9 to 133875.3) | 112(92.8 to 147.6) | -0.7(-0.8 to -0.6) |
| Qatar | 430.9(333.8 to 546.5) | 338.9(264.5 to 428.3) | 1286.1(955.2 to 1781.2) | 115(87.8 to 151.8) | -3.7(-4.3 to -3.1) |
| Saudi Arabia | 10336.2(7281.4 to 15732.6) | 155(110.5 to 232.6) | 22106.3(16294.2 to 35375) | 85.6(66.7 to 131.2) | -2.0(-2.2 to -1.8) |
| Singapore | 9204.7(8675.1 to 9737) | 388.5(367.3 to 410.2) | 7794.7(7085.8 to 8467.4) | 92.4(84 to 100.5) | -4.6(-4.9 to -4.4) |
| South Korea | 489123(356245.5 to 549575.8) | 1435.4(1091.5 to 1613.8) | 260065.4(218480.7 to 330430.9) | 288.8(242.9 to 366.1) | -5.5(-5.6 to -5.3) |
| Sri Lanka | 25487(21106.9 to 29566.2) | 215.1(178 to 249.7) | 23982(15288.5 to 33268.8) | 88.1(56.5 to 121.8) | -3.1(-3.3 to -2.8) |
| Syrian Arab Republic | 11666.9(8931.7 to 14646.5) | 199(152.6 to 249.8) | 18631.8(13606.3 to 24805.3) | 137.1(102.1 to 180.2) | -1.4(-1.6 to -1.2) |
| Taiwan (Province of China) | 79573.2(69144.7 to 87024.9) | 469.6(407.3 to 515) | 79425(71167.9 to 87813.1) | 193.7(174.5 to 213.3) | -3.3(-3.5 to -3.1) |
| Tajikistan | 23199(18954.2 to 27236.8) | 784.1(643.8 to 923.6) | 24523.9(19039.7 to 32616) | 368.2(288.7 to 485.2) | -2.5(-2.6 to -2.3) |
| Thailand | 117152.3(82368.6 to 142948.2) | 293.1(205.9 to 356.3) | 202764.3(117040.4 to 270597.7) | 197.2(113.7 to 261) | -1.7(-1.9 to -1.5) |
| Timor-Leste | 945.7(629.8 to 1292.5) | 272(185.4 to 361.9) | 1668.5(1197.9 to 2228) | 185.9(133.8 to 248.8) | -1.2(-1.4 to -0.9) |
| Turkey | 240297.2(179010.5 to 283344.1) | 629.6(478.4 to 739.3) | 254282.9(186670.3 to 314735.2) | 267(196.5 to 329.9) | -2.9(-3.2 to -2.6) |
| Turkmenistan | 12443(11512.5 to 13400.4) | 590.6(545.1 to 637.8) | 11695.9(8982.3 to 15329) | 260.4(200.3 to 340.3) | -2.7(-3.0 to -2.4) |
| United Arab Emirates | 1990.3(1450.9 to 2661.9) | 350.2(260.5 to 458.3) | 6484.4(4961.7 to 8904.3) | 158.4(126.2 to 206.9) | -1.3(-1.8 to -0.9) |
| Uzbekistan | 70636.8(64660.1 to 76684.1) | 574.9(523.9 to 626.6) | 61964.5(49849.6 to 75389.6) | 207.3(166.7 to 251.2) | -3.0(-3.2 to -2.8) |
| Viet Nam | 165207.8(122343.5 to 213101) | 391.4(288.5 to 502.9) | 210211.8(157400.1 to 276307) | 196.8(150.4 to 257.7) | -2.6(-2.7 to -2.4) |
| Yemen | 35130.1(18480.6 to 49121.4) | 634.9(340.7 to 874.8) | 73611.7(31019.7 to 106548.1) | 464.6(201.3 to 660.2) | -1.2(-1.3 to -1.1) |

ASDR: Age standardized DALYs rate. EAPC: Estimated annual percentage change. UI: Uncertain interval. CI: Confidence interval
