# Supplementary figures and images for: The Burden and Risk Factors of Gastric Cancer in Eastern Asia From 1990 to 2021: Longitudinal Observational Study of the Global Burden of Disease Study 2021
Source: JMIR Cancer. 2025 Aug 8;11:e75728. doi: 10.2196/75728 (PMC12334143; doi:10.2196/75728)

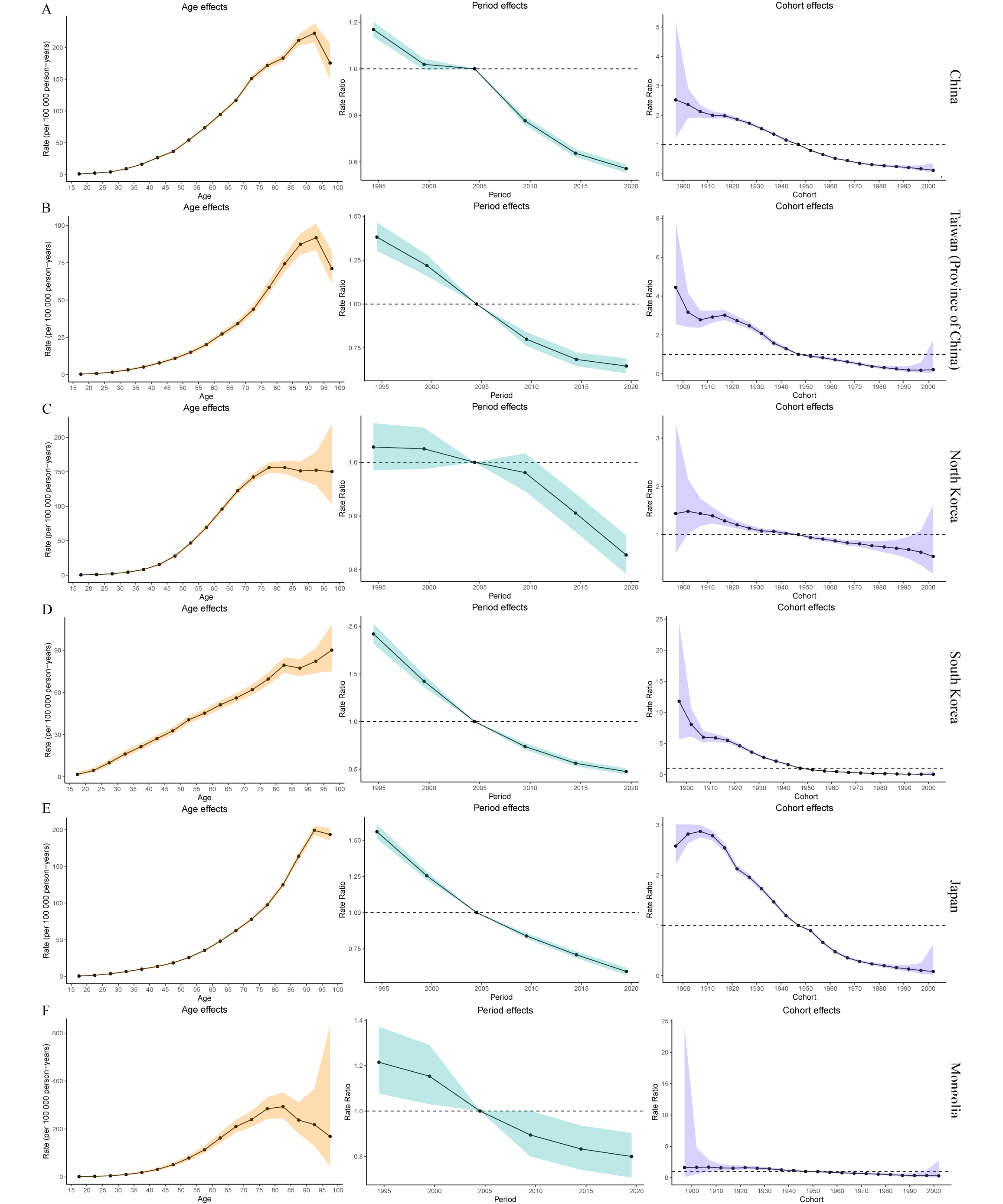

Supplement: Multimedia Appendix 4 [file cancer-v11-e75728-s004.png]

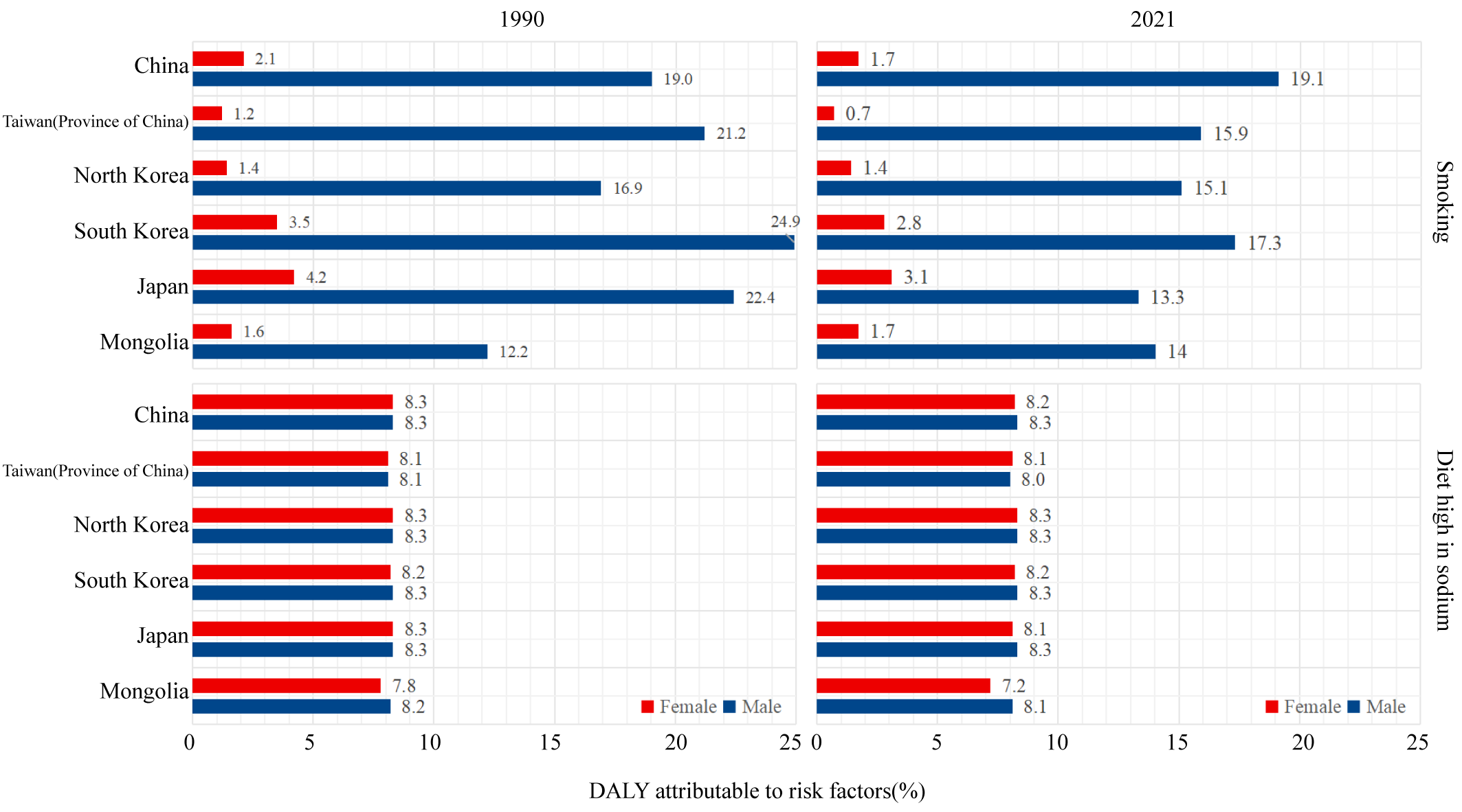

Supplement: Multimedia Appendix 5 [file cancer-v11-e75728-s005.png]

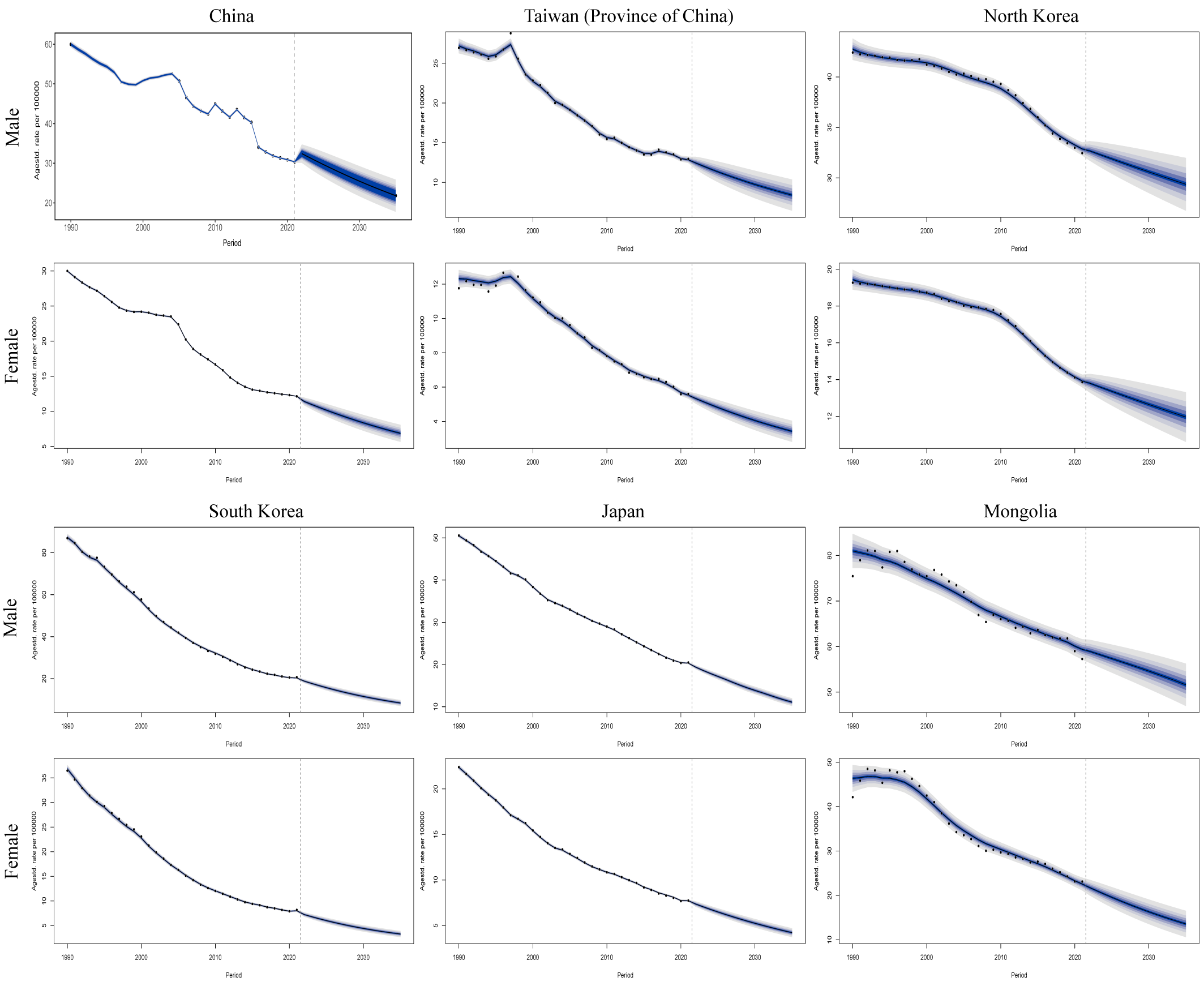

Supplement: Multimedia Appendix 6 [file cancer-v11-e75728-s006.png]
